# The skin-specific long non-coding RNA TEDAR orchestrates late epidermal differentiation via an ERK-KLF4 cytoplasmic regulatory axis

**DOI:** 10.1038/s41419-026-09192-0

**Published:** 2026-09-05

**Authors:** Kunal Das Mahapatra, Özge Arslan, Longlong Luo, Evelyn Kelemen, Sreeram Peringattu Kalarikkal, Lorenzo Pasquali, Christian Ziegler, Johannes Graf, Nicole Hemmer, Rachael Sugars, Markus Kretz, Eniko Sonkoly, Andor Pivarcsi

**Affiliations:** 1 Department of Medical Biochemistry and Microbiology, Uppsala University, Uppsala, Sweden; 2 Department of Medical Sciences, Uppsala University, Uppsala, Sweden; 3 Department of Medicine Solna, Karolinska Institutet, Stockholm, Sweden; 4 Regensburg Center for Biochemistry (RCB), University of Regensburg, Regensburg, Germany; 5 Eberhard-Karls-Universität Tübingen, Geschwister-Scholl-Platz, Tübingen, Germany; 6 Department of Dental Medicine, Karolinska Institutet, Stockholm, Sweden; 7 Institute for Molecular Medicine, MSH Medical School Hamburg, Hamburg, Germany

**Keywords:** Cell biology, Differentiation, Atopic dermatitis, Chronic inflammation

## Abstract

The formation of a functional skin barrier depends on terminal keratinocyte differentiation, yet the contribution of long non-coding RNAs to this process remains incompletely understood. Here, using transcriptome analysis of more than 15,000 RNA-seq samples across 54 human tissues, we identify *TEDAR* (Terminal Epidermal Differentiation-Associated RNA), a highly skin-enriched lncRNA. Single-molecule RNA in situ hybridization revealed that *TEDAR* is confined to the uppermost granular layer of the epidermis, representing an exceptionally spatially restricted expression pattern. Functional studies demonstrated that CRISPR-mediated activation of *TEDAR* promotes keratinocyte differentiation even in the absence of external cues, while its depletion impaired late epidermal differentiation. In three-dimensional skin equivalents, *TEDAR* depletion severely compromises stratum corneum formation, highlighting its importance for proper epidermal barrier assembly. Mechanistically, *TEDAR* associates with ERK1/2-containing complexes and restrains ERK phosphorylation, thereby facilitating the nuclear accumulation of KLF4, a key regulator of epidermal differentiation. KLF4 depletion attenuated TEDAR-induced expression of several late differentiation genes, supporting a functional TEDAR–ERK–KLF4 axis. Clinically, *TEDAR* expression was reduced in cutaneous squamous cell carcinoma (cSCC) and chronic inflammatory skin diseases, including psoriasis and atopic dermatitis. Moreover, IL-22 suppressed *TEDAR* via STAT3 signalling in epidermal models. These findings identify *TEDAR* as an important regulator of terminal epidermal differentiation and suggest that its suppression may contribute to disturbed differentiation in inflammatory and neoplastic skin diseases.

## Introduction

The epidermis is a stratified epithelium that forms the body’s primary barrier against dehydration, mechanical trauma, and invasion by pathogenic microorganisms. This barrier is established through the complex and tightly regulated program of keratinocyte terminal differentiation in which basal progenitors exit the cell cycle and undergo a highly ordered terminal differentiation program to generate the cornified envelope of the stratum corneum [1]. The regulation of terminal differentiation in the epidermis is a central process in skin homeostasis, and its impairment is associated with a broad range of genetic, inflammatory and malignant skin diseases [2–6]. While transcription factors, signaling pathways, and structural proteins regulating epidermal differentiation have been extensively characterized over the past decades, the contribution of the non-coding genome to this process has remained incompletely defined [7–9].

Long non-coding RNAs (lncRNAs) are a structurally and functionally diverse group of transcripts defined as > 500 nucleotides with minimal or no protein-coding capacity [10]. Many lncRNAs are expressed in a tissue-, developmental stage- and species-specific manner, suggesting pivotal roles in defining cell identity and organismal complexity [11–13]. Mechanistically, lncRNAs can regulate gene expression by interacting with transcription factors, epigenetic modifiers in the nucleus, or by interacting with signaling and RNA-protein complexes to modulate post-transcriptional control and signal transduction [14–16]. Despite their importance in development and diseases, their role in epidermal differentiation remains largely unexplored and debated [17].

The transcript *LINC00302* (historically annotated as *XP33, ELDAR*) was independently described by other groups and us as skin-enriched [18–21]. However, its spatial expression, function, molecular mechanism of action, and its regulation in skin diseases have remained unresolved.

Here, we define the function and mechanism of this highly keratinocyte-specific, differentiation-induced lncRNA, which we term TEDAR (*Terminal Epidermal Differentiation-Associated RNA*). We show that TEDAR expression is sharply restricted to the upper granular layer of keratinizing epidermis. Functionally, TEDAR is required for late-stage differentiation and proper stratum corneum ultrastructure. Mechanistically, TEDAR associates with the ERK1/2 signaling complex to restrain ERK phosphorylation, thereby enabling KLF4 nuclear accumulation and activation of terminal differentiation gene programs. Finally, we identify a disease-relevant regulatory axis in which IL-22/STAT3 signaling suppresses TEDAR, linking inflammatory signaling to impaired differentiation in psoriasis and atopic dermatitis, and to TEDAR loss in cutaneous squamous cell carcinoma.

## Materials and methods

### Clinical samples and cell culture

Skin biopsies were obtained from healthy donors and patients with atopic dermatitis, psoriasis, or cutaneous squamous cell carcinoma (cSCC) at the Karolinska University Hospital, Sweden, following informed consent and ethical approval. The study was approved by the Swedish Ethical Review Authority and conducted according to the Declaration of Helsinki. Normal human epidermal keratinocytes (NHEKs) were cultured in EpiLife medium and differentiated by adding 1.5 mM CaCl_2_. cSCC cell lines were cultured in DMEM with 10% FBS. 3D organotypic epidermal equivalents were generated by seeding keratinocytes onto polycarbonate inserts, submerged for 48 h, and then maintained at the air-liquid interface for 4 days.

### Genetic manipulation and treatments

For knockdown, NHEKs were electroporated with 1 nmol siPools targeting *TEDAR* or non-targeting controls (siTOOLs Biotech). For transcriptional activation (CRISPRa), cells were transfected with sgRNA (MS2) cloning backbone, MS2-P65-HSF1-GFP, and dCas9-VP64-GFP plasmids (Addgene). Cytokine treatments (e.g., IL-22, IL-17A) and STAT3 inhibition (C188-9, WP1066) were performed as detailed in the Supplementary Information.

### Expression and interaction analysis

Gene expression was quantified by qRT-PCR using SYBR Green and normalized to *18S* rRNA. Primers are listed in Supplementary Table S8. Protein expression was analyzed by Western blotting using antibodies listed in Supplementary Table S9. Subcellular fractionation was performed to isolate nuclear and cytoplasmic compartments. *TEDAR*-protein interactions were investigated via RNA pull-down using biotinylated transcripts followed by mass spectrometry, and by RNA Immunoprecipitation (RIP) using anti-ERK1/2 antibodies.

### Imaging and histology

Single-molecule RNA in situ hybridization (RNAscope, ACD) and immunofluorescence were performed on FFPE tissue sections or fixed cells. The ultrastructure of 3D skin models was analyzed by Transmission Electron Microscopy (TEM).

### Bioinformatics and statistics

Skin-enriched transcripts were identified using GTEx RNA-seq data in R. Differential expression in *TEDAR*-depleted models was profiled using Affymetrix HuGene-2.1 arrays. Public RNA-seq datasets (GSE121212, GSE274560) were analyzed for disease associations. Data are presented as mean ± SEM. Statistical significance was determined using Student’s *t*-test, ANOVA, or non-parametric tests as appropriate (*P* < 0.05). For detailed descriptions of bioinformatics pipelines and statistical approaches, see Supplementary Materials and Methods.

Detailed protocols and full-size Western blot images are provided as Supplementary Material.

## Results

### TEDAR is a keratinizing skin-enriched lncRNA with granular-layer specificity in vivo

**Fig. 1:**
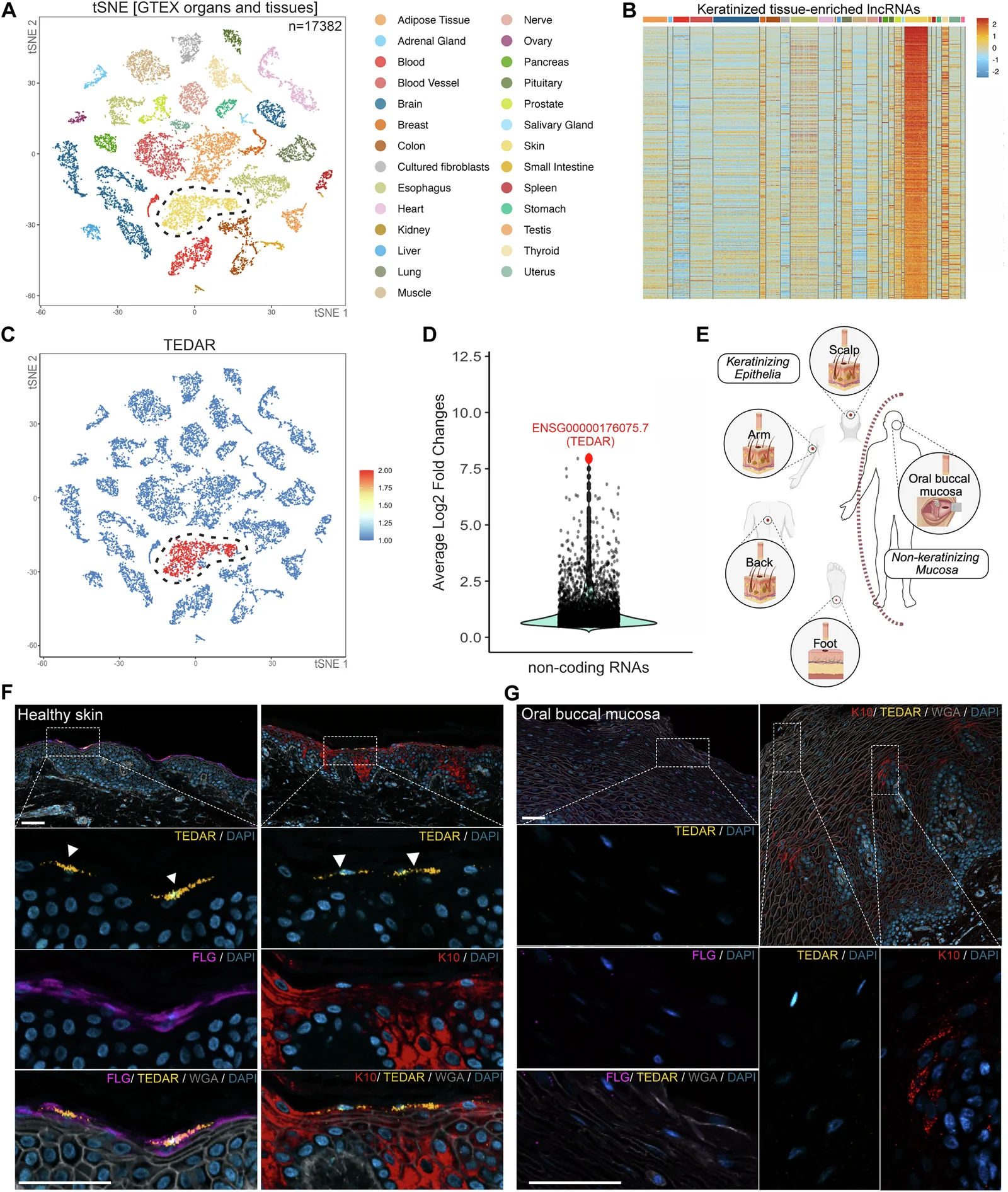
TEDAR is a skin-enriched suprabasal lncRNA in keratinizing epidermis. **A** t-SNE visualization of 17,382 tissue samples from the GTEx database. **B** Heatmap of the top 500 skin-enriched lncRNAs. **C** Feature plot of *TEDAR* expression across GTEx tissues. **D** Fold enrichment of non-coding RNAs across tissues; *TEDAR* is highlighted in red. **E** Schematic of biopsy sites comparing keratinizing epidermis (skin) vs. non-keratinizing epithelium (buccal mucosa). **F** Single-molecule in situ hybridization (RNAscope) of *TEDAR* (yellow) with immunofluorescence for Filaggrin (FLG, magenta) and Keratin 10 (KRT10, red) in healthy skin. Nuclei are counterstained with DAPI (blue); WGA staining (gray) delineates cell boundaries. Arrowheads indicate *TEDAR* signal in the granular layer (*n* = 6). Scale bars, 50 µm. **G** Immunofluorescence for FLG (magenta) and KRT10 (red) in combination with RNAscope of *TEDAR* in buccal mucosa showing minimal expression. Scale bars, 50 µm.

To identify lncRNAs with potential roles in epidermal differentiation, we analyzed transcriptomic data from 17,382 tissue samples across 54 non-diseased human tissues and organs generated by the Genotype-Tissue Expression (GTEx) project. Dimensionality reduction (t-SNE) distinctively separated skin samples from other tissues, enabling robust identification of skin-enriched mRNAs and non-coding RNAs (Fig. 1A, B and Supplementary Fig. S1A; Tables S1, S2). Consistent with expected biology, skin-enriched mRNAs were strongly enriched for processes intrinsic to skin biology, including epidermal development, keratinocyte differentiation, and tight junction assembly (Supplementary Fig. S1B).

Within the skin-enriched non-coding signature, *ENSG00000176075.7* (*TEDAR*) emerged as the most highly enriched lncRNA, being abundantly expressed in the skin while virtually undetectable across other human tissues (Fig. 1C, D and Supplementary Fig. S1C, D). qRT-PCR analysis across major skin-resident cell types revealed that *TEDAR* expression is restricted to differentiated keratinocytes (Supplementary Fig. S1E).

To determine the spatial distribution of *TEDAR* in vivo, we performed single-molecule RNA in situ hybridization (RNAscope) on human skin biopsies from multiple anatomical sites (Fig. 1E). Strikingly, *TEDAR* expression was confined to a single cell layer in the healthy human epidermis, specifically the uppermost granular layer of the epidermis, overlapping with the late differentiation marker filaggrin (*FLG*) but distinct from the spinous layer marker keratin 10 (*KRT10*) (Fig. 1F and Supplementary Fig. S1F, G). Notably, analysis of GTEx RNA-seq data revealed that *TEDAR* is absent from non-keratinizing squamous epithelia, including the vagina, esophageal mucosa, and bladder, while being abundantly expressed in the skin (Supplementary Fig. S1H). This was further corroborated by RNAscope, which confirmed that *TEDAR* is virtually absent in the non-keratinizing buccal mucosa (Fig. 1G). Together, these data identify *TEDAR* as a highly spatially restricted, keratinizing-epithelium-associated lncRNA consistent with a role in late-stage epidermal differentiation.

### *TEDAR* is a non-coding transcript with strong primate sequence conservation that localizes predominantly to the cytoplasm

**Fig. 2:**
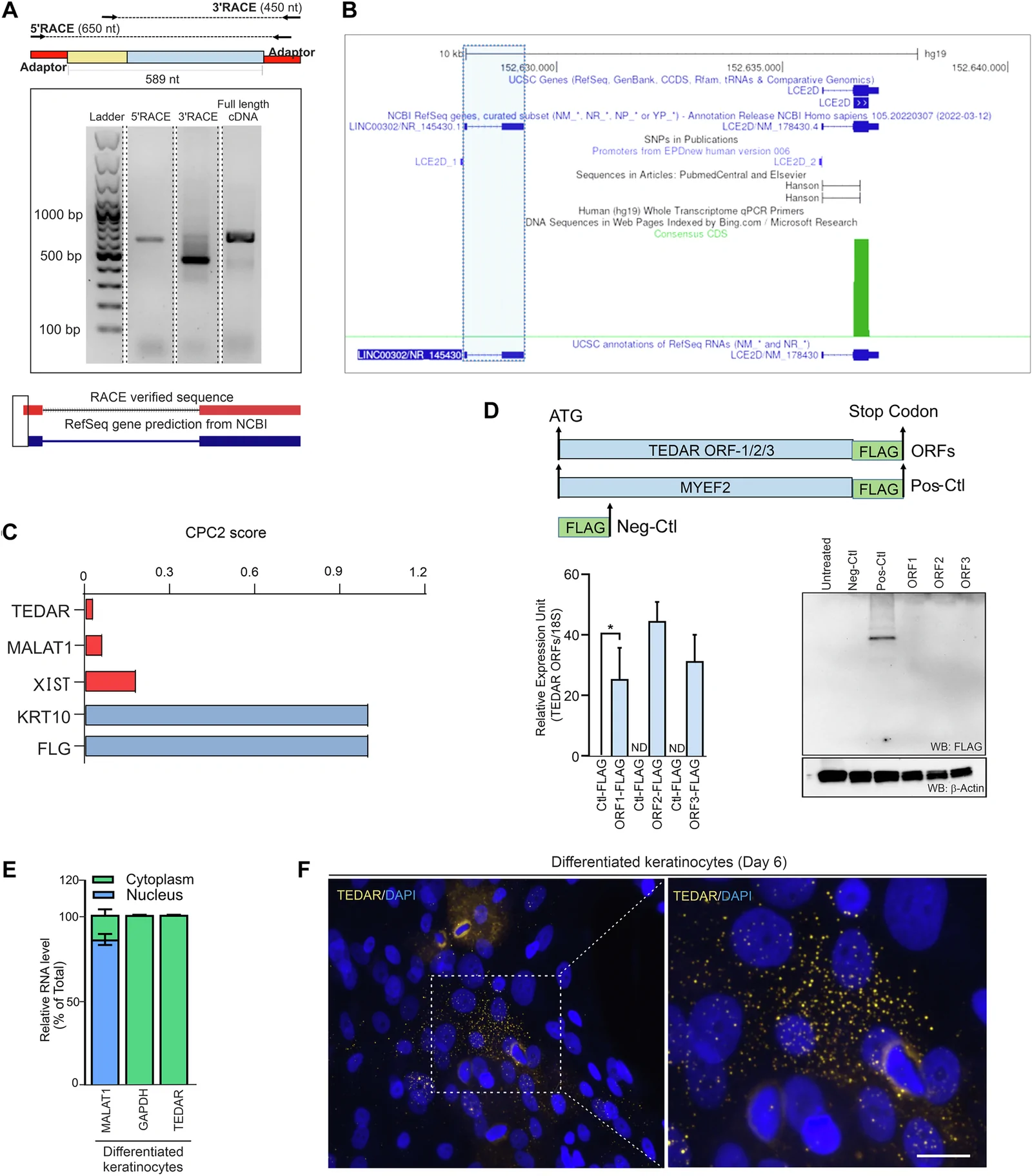
TEDAR is a long non-coding RNA with cytoplasmic localization in differentiated keratinocytes. **A** Schematic of 5′ and 3 RACE identifying the 589-nt *TEDAR* transcript. Gel shows products from differentiated keratinocytes (*n* = 2). **B** UCSC genome browser view of the *TEDAR* (*LINC00302*) locus within the Epidermal Differentiation Complex. **C** Coding Potential Calculator (CPC2) score of *TEDAR* compared to non-coding (*MALAT1*, *XIST*) and coding (*KRT10*, *FLG*) controls. **D** Top: Schematic of *TEDAR* ORF constructs. Bottom: Western blot using anti-FLAG antibody in keratinocytes transfected with *TEDAR* ORFs or positive control (*n* = 3). **E** qRT-PCR of *TEDAR* in nuclear vs. cytoplasmic fractions of differentiated keratinocytes (*n* = 3). **F** RNAscope showing cytoplasmic *TEDAR* localization (yellow) in differentiated keratinocytes. Scale bar, 50 µm. Data are mean ± SEM.

*TEDAR* maps to the 1q21.3 region within the Epidermal Differentiation Complex (EDC), positioned between two major late cornified envelope (*LCE*) gene clusters (*LCE2* and *LCE3*) (Supplementary Fig. S2A). Using Rapid Amplification of cDNA Ends (RACE), we identified a single transcript of 589 nucleotides in differentiated keratinocytes consisting of two transcribed exons (Fig. 2A, B). Analysis of the locus conservation using PhastCons tracks revealed that the *TEDAR* sequence and exon structure are highly conserved among primates but show limited conservation in rodents (PhastCons; Supplementary Fig. S2D).

Although *TEDAR* corresponds to a locus historically annotated as a putative protein-coding gene, *XP33* [18], our data support its classification as a non-coding RNA. Bioinformatic analysis (CPC2) yielded a low coding potential score (Fig. 2C). Furthermore, we found no evidence of xp33 peptides in public skin proteomics datasets [22], and the expression of predicted *TEDAR* open reading frames (ORFs) failed to produce detectable peptides in keratinocytes (Fig. 2D). Subcellular fractionation and RNAscope analysis demonstrated that *TEDAR* is predominantly localized to the cytoplasm of differentiated keratinocytes (Fig. 2E, F). Consistent with recent reports identifying this transcript (*LINC00302*) as differentiation-associated [21], we confirmed that *TEDAR* is virtually undetectable in undifferentiated cells but is strongly induced during the late stages of differentiation (days 4-7), paralleling the induction of *FLG* and *LOR* (Supplementary Fig. S2A–C). Together, these data define *TEDAR* as a differentiation-induced, non-coding, cytoplasmic transcript encoded within the EDC and exhibiting strong primate sequence conservation.

### Transcriptional activation of TEDAR promotes late-stage keratinocyte differentiation

**Fig. 3:**
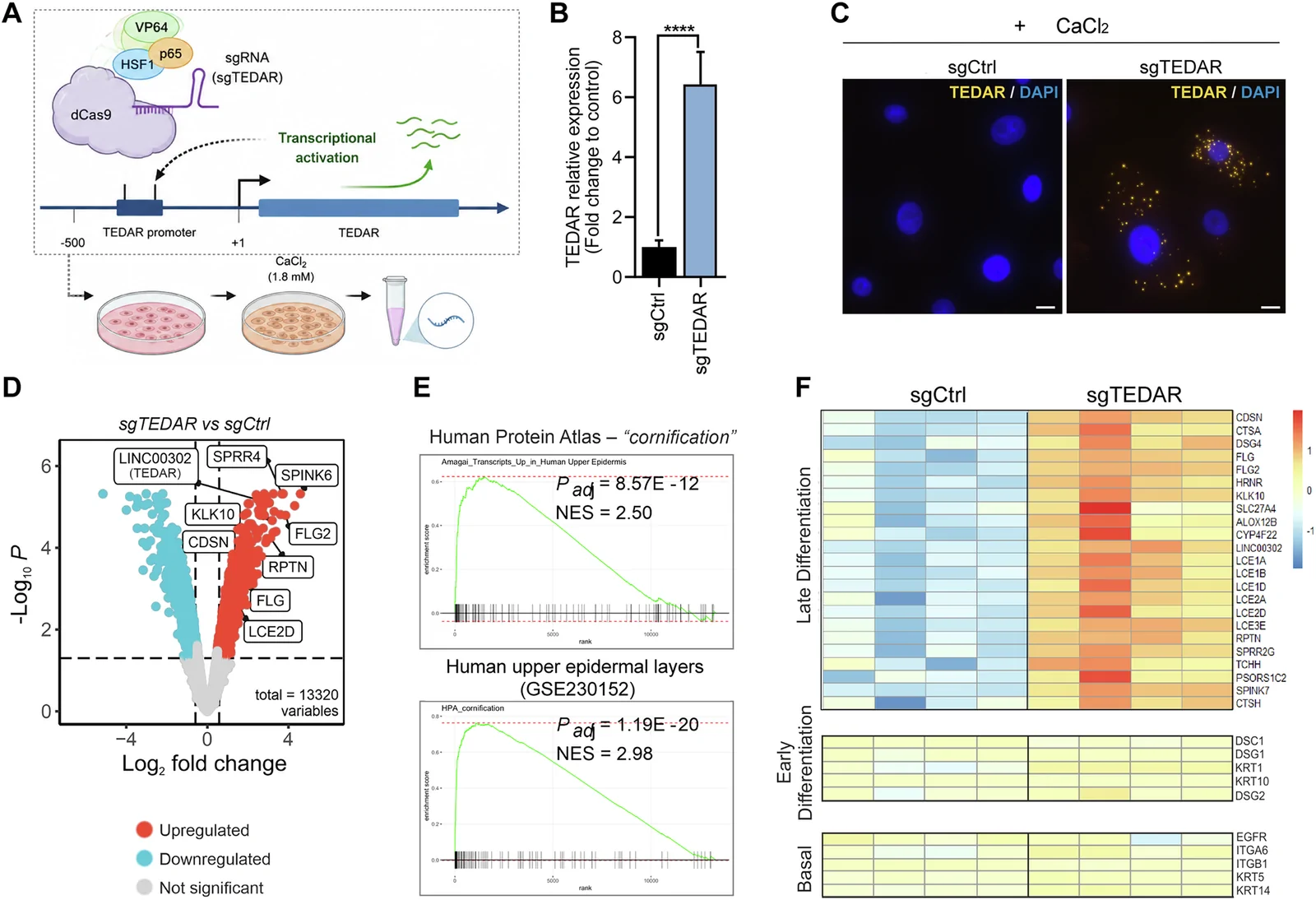
Activation of *TEDAR* promotes epidermal differentiation. **A** Schematic of CRISPR-activation (CRISPRa) targeting the *TEDAR* promoter in NHEKs. **B** qRT-PCR confirmation of *TEDAR* upregulation (*n* = 3). **C** RNAscope showing increased *TEDAR* levels in CRISPRa cells. **D** Volcano plot of differentially expressed genes following *TEDAR* activation. **E** GSEA enrichment plots for “Cornification” (Human Protein Atlas) and “Upper Epidermis” signatures. **F** Heatmap of differentiation-associated genes. Data are mean ± SEM. *P < 0.05, **P < 0.01, ***P < 0.001, ****P < 0.0001 (Student’s *t*-test).

To test whether *TEDAR* functions as a regulator of differentiation, we transcriptionally activated the endogenous locus in primary human keratinocytes using the CRISPR-Cas9 Synergistic Activation Mediator (SAM) system (Fig. 3A). To achieve CRISPR-SAM-mediated TEDAR activation (CRISPRa), four sgRNAs targeting the proximal TEDAR promoter were screened; sgRNA 2 produced robust and reproducible TEDAR induction ( ~ 60-fold; Supplementary Fig. S3A, B) and was selected for all subsequent experiments (Fig. 3A, B). CRISPR activation (CRISPRa) resulted in a significant upregulation of *TEDAR*, with maintained cytoplasmic localization (Fig. 3B, C). Transcriptomic profiling revealed broad induction of cornification- and upper-epidermis-associated gene programs following TEDAR activation (Fig. 3D and Supplementary Table S3). qRT-PCR analysis confirmed that *TEDAR* activation significantly upregulated late differentiation markers (*FLG*, *LOR*, *LCE2D*, *LCE3A*) both in the absence and presence of calcium without altering the early marker *KRT10* (Supplementary Fig. S3C, D). Gene Ontology (GO) term enrichment and Gene Set Enrichment Analysis (GSEA) further supported the induction of multiple epidermal differentiation-related processes including the GO terms “epidermis development” (GO: 0008544) and “epidermal cell differentiation” (GO:0009913)) and keratinocyte differentiation gene sets such as “epidermis development” and “keratinocyte differentiation”, “Human Protein Atlas-cornification”, and genes expressed in the upper epidermal layers [23] (Fig. 3E, Supplementary Fig. S3E, F and Supplementary Table S4). In addition to structural proteins (e.g. LCEs, CDSN, SPRRs, FLG, FLG2), *TEDAR* activation regulated several families of enzymes, transporters critical for barrier integrity and lipid-processing enzymes (e.g. ALOX12B), required for the lipid barrier. Importantly, basal layer-associated genes (e.g. KRT5, KRT14) or early suprabasal layer genes (e.g. KRT10) were unaffected (Fig. 3F), indicating a specific role for TEDAR in promoting late epidermal differentiation. Taken together, these findings establish TEDAR as a potent and selective driver of late keratinocyte differentiation programs.

### TEDAR is required for terminal differentiation and proper stratum corneum ultrastructure

**Fig. 4:**
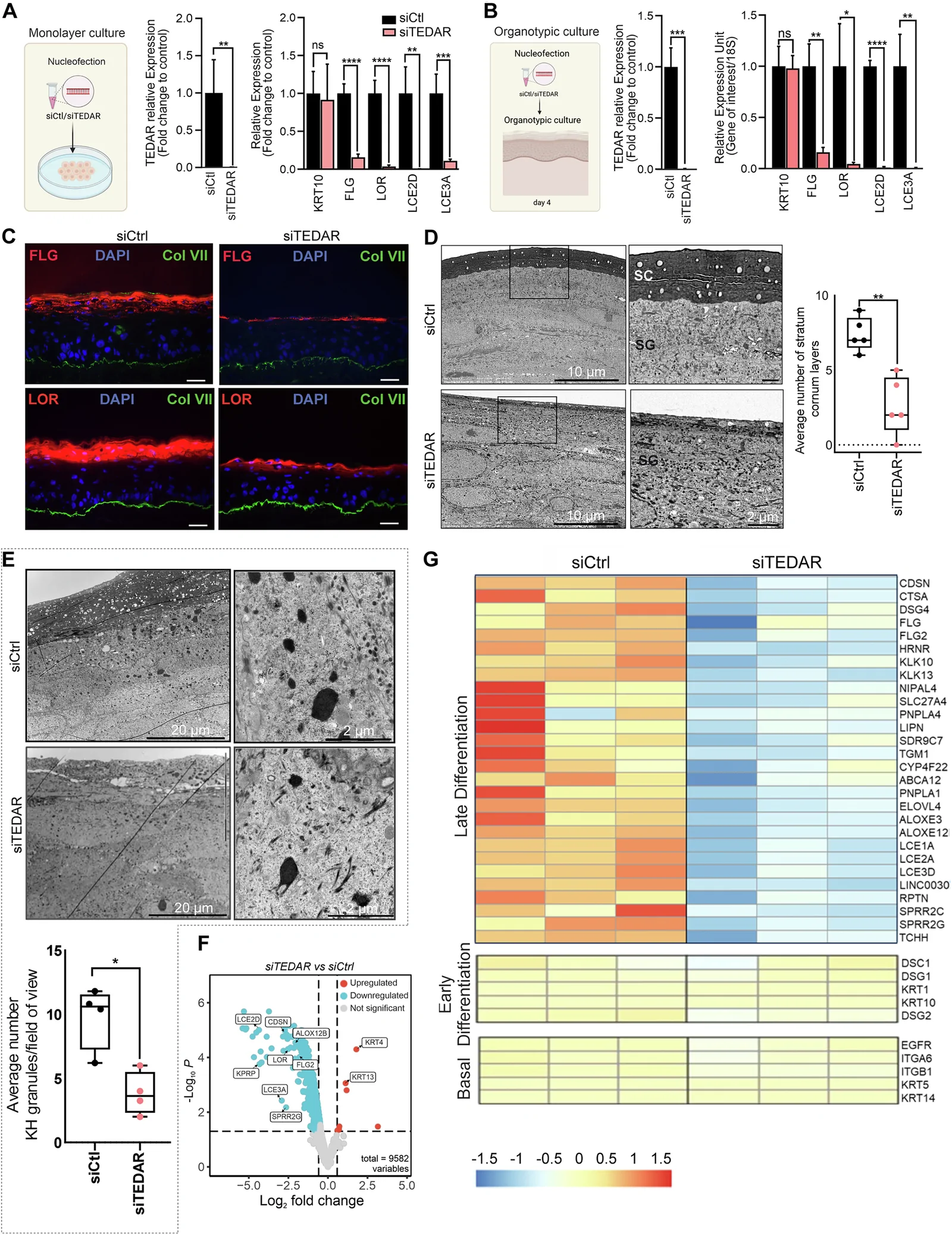
*TEDAR* is required for terminal keratinocyte differentiation and skin barrier formation. **A** qRT-PCR of differentiation markers in monolayer keratinocytes transfected with siControl or siTEDAR (*n* = 3). **B** qRT-PCR of differentiation markers in 3D organotypic skin equivalents (*n* = 3). **C** Immunofluorescence of Filaggrin (FLG) and Loricrin (LOR) in 3D skin models. Green: Collagen VII (basement membrane). Scale bars, 50 µm. **D** Transmission electron microscopy (TEM) of stratum corneum (SC) and stratum granulosum (SG) in siControl and siTEDAR 3D skin models. Right: Quantification of the average number of SC layers per field of view (*n* = 5). Scale bars: 10 µm (low magnification), 2 µm (high magnification). *P < 0.01 (Student’s *t*-test). **E** TEM images showing keratohyalin (KH) granules in the stratum granulosum of siControl and siTEDAR 3D skin models. Bottom: Quantification of the average number of KH granules per field of view (*n* = 5). Scale bars, 20 µm (low magnification) and 2 µm (high magnification). *P < 0.05 (Student’s *t*-test). **F** Volcano plot of transcriptomic profiling data from *TEDAR*-depleted 3D skin models. **G** Heatmap of differentiation markers. Data are mean ± SEM. *P < 0.05, **P < 0.01, ***P < 0.001 (Student’s *t*-test).

To assess the physiological necessity of *TEDAR*, we performed loss-of-function experiments. In monolayer keratinocytes, *TEDAR* knockdown significantly impaired the induction of late differentiation markers (*FLG*, *LOR*, *LCE2D*, *LCE3A*) upon calcium treatment while leaving the early differentiation marker KRT10 unaffected, indicating a specific defect in the transition to the granular state (Fig. 4A).

To assess the role of TEDAR in a more physiological setting, we next constructed 3D organotypic skin equivalents using *TEDAR*-depleted keratinocytes (Fig. 4B). Consistent with the monolayer data, *TEDAR* knockdown led to a marked reduction in late differentiation gene expression (Fig. 4B) and a visible decrease in Filaggrin and Loricrin protein levels (Fig. 4C). Strikingly, transmission electron microscopy (TEM) revealed severe ultrastructural defects: *TEDAR*-depleted tissues displayed a significantly thinner stratum corneum and a reduced number of keratohyalin granules, the cytoplasmic reservoirs for profilaggrin (Fig. 4D, E). Importantly, these structural defects occurred without detectable changes in proliferation or apoptosis (Supplementary Fig. S4).

Transcriptomic profiling of *TEDAR*-depleted 3D skin equivalents identified widespread repression of gene programs associated with cornification and upper-epidermal differentiation with a mild upregulation of *KRT4* and *KRT13*, keratins that are primarily expressed in the suprabasal layers of non-cornified stratified squamous epithelia (Fig. 4F and Supplementary Table S5). The repressed programs included GO terms and GSEA gene sets related to “cornified envelope”, “keratinization”, and “upper epidermis” (Supplementary Fig. S5A, B)and Supplementary Table S6. Consistently, the transcriptomes from TEDAR-depleted 3D skin equivalents showed strong negative enrichment for genes expressed highly in the upper epidermis as defined by Tahara et al. (GSE230152) [23] and suprabasal keratinocyte-associated according to the Human Protein Atlas (Supplementary Fig. S5C). Notably, TEDAR depletion reduced the expression of essential barrier genes, including *FLG, FLG2, HRNR, RPTN*, SPRR family members, and desquamation-associated kallikreins (e.g. *KLK10)* as well as corneodesmosin (Fig. 4G and Supplementary Fig. S5D, E). In contrast, no changes were observed in progenitor (e.g. *KRT14, KRT5*) or early differentiation markers (*KRT1, KRT10*) (Fig. 4G). Intersection of the CRISPRa and siRNA datasets identified a core set of 118 concordantly *TEDAR*-regulated genes, highly enriched for cornified envelope formation pathways (Supplementary Fig. S6). Collectively, these results demonstrate that *TEDAR* is required for the proper formation of the cornified envelope in human epidermal models.

### TEDAR regulates the nuclear accumulation of KLF4, a key driver of squamous differentiation

**Fig. 5:**
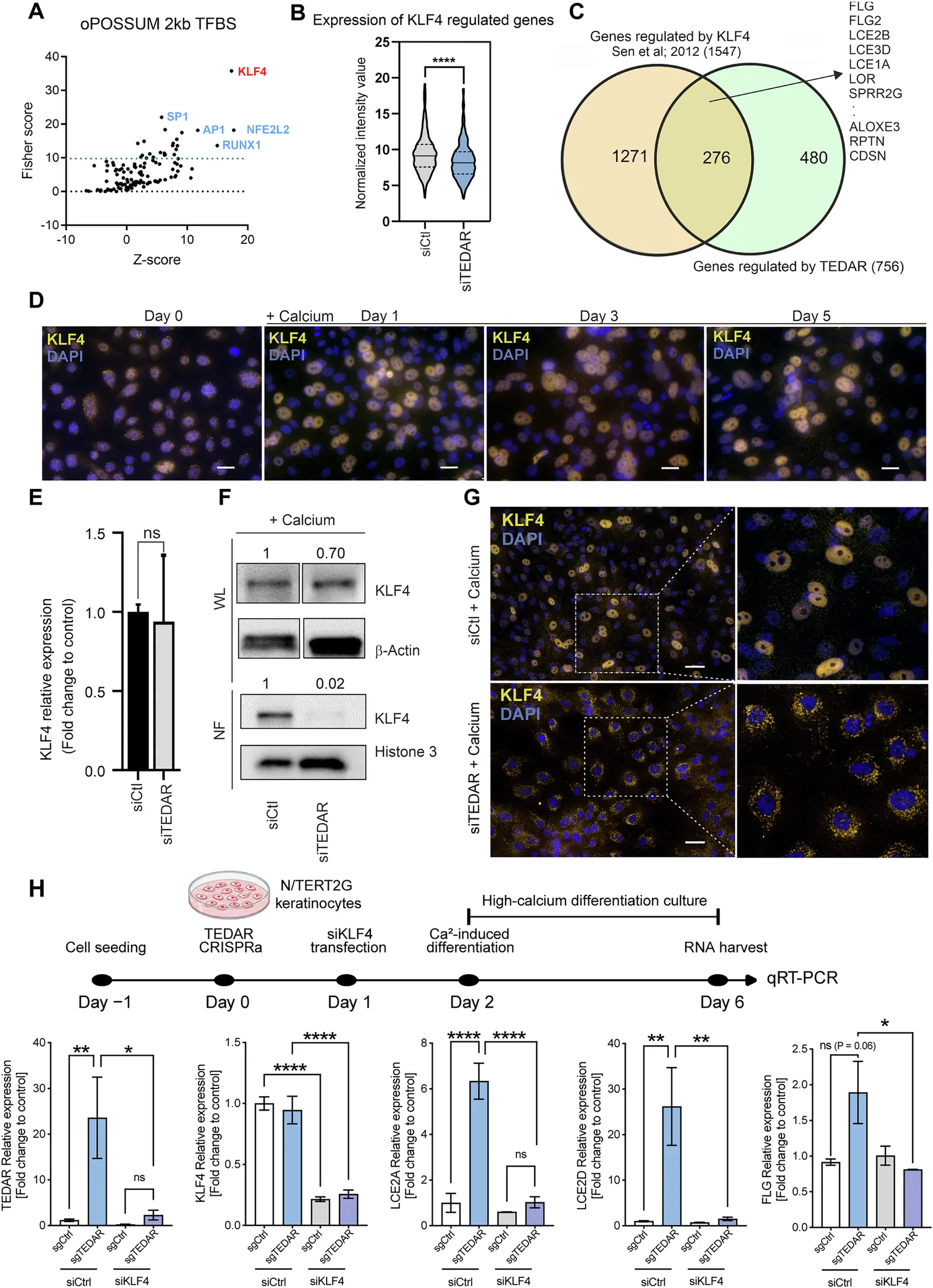
*TEDAR* is required for the nuclear localization of KLF4 during keratinocyte differentiation. **A** oPOSSUM analysis identifying KLF4 binding motifs as highly enriched in *TEDAR*-regulated genes. **B** Violin plot of normalized expression for KLF4-target genes upon *TEDAR* depletion. **C** Venn diagram of overlap between *TEDAR*-regulated genes and the KLF4 regulon. **D** Immunofluorescence of KLF4 translocation during differentiation (*n* = 3). **E** qRT-PCR of *KLF4* mRNA in siControl vs. siTEDAR cells (*n* = 3). **F** Western blot of KLF4 in whole lysate (WL) vs. nuclear fraction (NF). Representative of three independent biological replicates; densitometric ratios normalized to β-actin or Histone H3 are indicated. **G** Immunofluorescence of KLF4 (yellow) in differentiated keratinocytes upon *TEDAR* depletion. Scale bars, 50 µm. Data are mean ± SEM. ****P < 0.0001 (Wilcoxon rank-sum test for (**B**), Student’s *t*-test for (**E**)). **H** Experimental design and qRT-PCR analysis of TEDAR activation combined with KLF4 depletion during calcium-induced keratinocyte differentiation. N/TERT2G keratinocytes were subjected to TEDAR CRISPRa, followed by siKLF4 transfection and high-calcium differentiation. TEDAR, KLF4, LCE2A, LCE2D, and FLG expression was analyzed on day 6. Data are shown as mean ± SEM. *P < 0.05, **P < 0.01, ***P < 0.001 (one-way ANOVA).

To identify the molecular mechanism through which *TEDAR* drives epidermal differentiation, transcription factor motif enrichment analysis was performed on *TEDAR*-regulated genes. This analysis using the oPOSSUM platform identified Krüppel-like factor 4 (KLF4), a master regulator of barrier formation [24, 25], as the most overrepresented factor (z-score = 17.32) (Fig. 5A). In line with this, TEDAR depletion was associated with reduced expression of KLF4 target gene sets and overlap with published KLF4-regulated genes (Fig. 5B, C), suggesting that TEDAR regulates terminal differentiation at least in part through KLF4-dependent transcriptional programs.

While the essential role of KLF4 in barrier formation is well-established [24, 25], its nucleo-cytoplasmic dynamics during keratinocyte differentiation remain incompletely defined. We therefore first examined KLF4 localization in normal primary keratinocytes during calcium-induced differentiation. We observed that KLF4 is predominantly cytoplasmic in proliferating keratinocytes but accumulated in the nucleus following calcium-induced differentiation (Fig. 5D). We next asked whether TEDAR regulates this process. Notably, TEDAR silencing did not affect total KLF4 mRNA levels (Fig. 5E) or total KLF4 protein expression in whole cell lysates (Fig. 5F); instead, it led to a strikingly reduced KLF4 accumulation in the nuclear fraction and diminished nuclear KLF4 signal by immunofluorescence (Fig. 5F, G), coinciding with reduced expression of KLF4 target genes (*FLG, LOR* and *TCHH)*. These findings indicate that *TEDAR* is required for efficient nuclear accumulation of KLF4 during keratinocyte terminal differentiation.

To functionally determine whether KLF4 is required downstream of TEDAR to drive epidermal differentiation, we performed CRISPRa-mediated TEDAR activation in combination with siRNA-mediated KLF4 depletion using N/TERT2G immortalized keratinocytes (Fig. 5H). Similar to primary keratinocytes, the activation of TEDAR in control cells successfully induced the expression of late differentiation markers (LCE2A, LCE2D, LCE1B and FLG; Fig. 5H and Supplementary Fig. S7A, B). We observed that knockdown of KLF4 following TEDAR-induction significantly blunted this induction for LCE2A, LCE2D and FLG, but not for LCE1B, suggesting that TEDAR can mediate its effect through more than one downstream pathway. Interestingly, knockdown of KLF4 resulted in decreased TEDAR-induction upon CRISPR-activation, suggesting a possible feedback loop between TEDAR and KLF4. These data suggest that TEDAR-mediated induction of the terminal differentiation program depends, at least in part, on the presence of KLF4, reinforcing the mechanistic link between TEDAR activity and KLF4-driven gene expression.

### TEDAR associates with ERK1/2 to restrain ERK phosphorylation and enable KLF4 nuclear accumulation

**Fig. 6:**
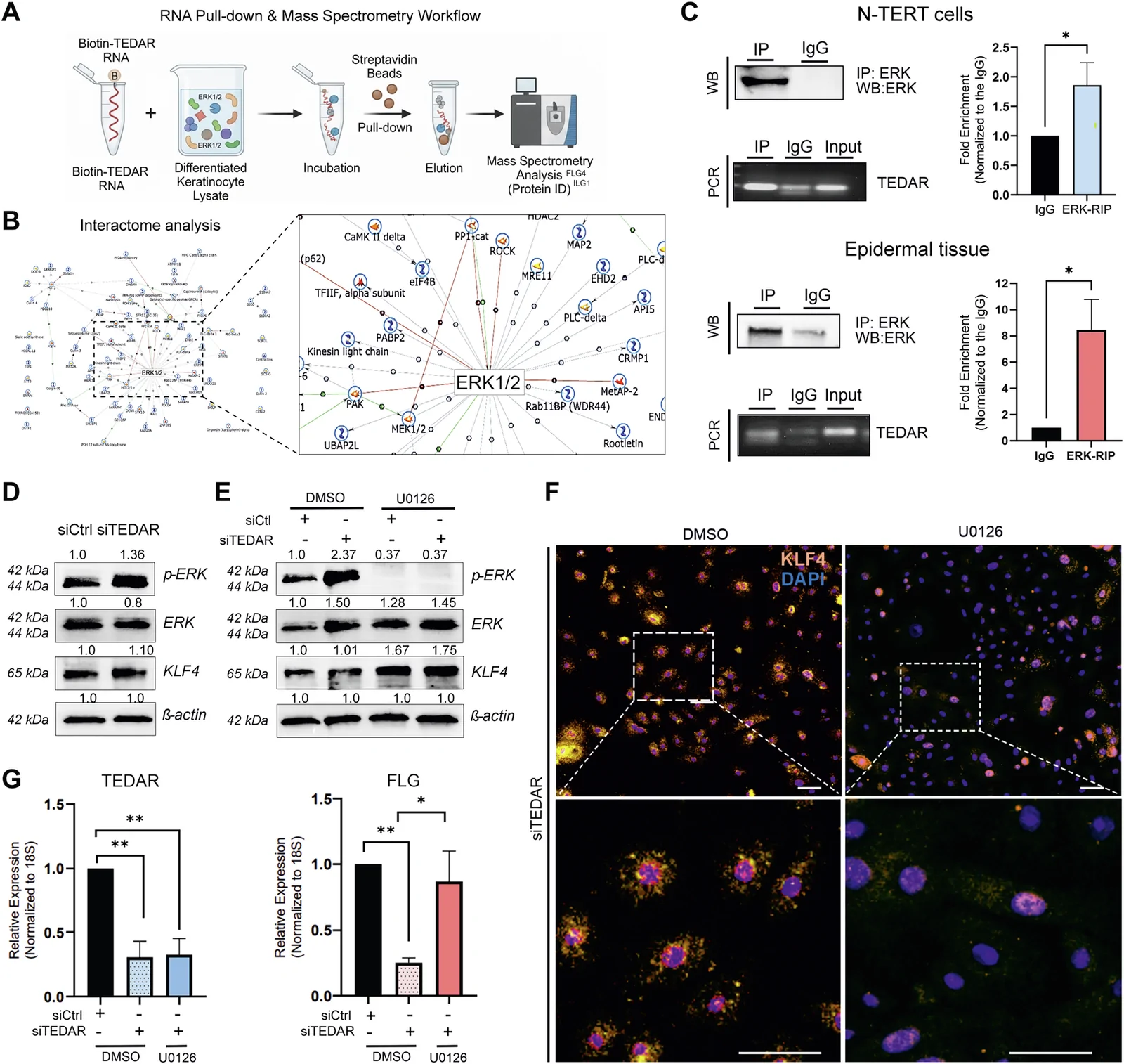
*TEDAR* associates with ERK1/2-containing complexes and modulates ERK phosphorylation to control KLF4 nuclear localization and keratinocyte differentiation. **A** Workflow for *TEDAR* RNA pull-down and mass spectrometry. **B** Network analysis of *TEDAR*-interacting proteins. **C** RNA immunoprecipitation (RIP) showing *TEDAR* enrichment with anti-ERK1/2 in N/TERT2G cells and epidermal tissue (*n* > 3). **D** Immunoblot of p-ERK, total ERK, and KLF4 in siControl vs. siTEDAR cells. **E** Immunoblot of p-ERK following treatment with MEK inhibitor U0126. Representative immunoblots of three independent biological replicates are shown; densitometric ratios normalized to β-actin are indicated. **F** Immunofluorescence of KLF4 (yellow) in siTEDAR cells treated with DMSO or U0126. Scale bars, 50 µm. **G** qRT-PCR analysis of TEDAR (left) and FLG (right) expression by U0126 in *TEDAR*-depleted cells (*n* = 3). Data are presented as mean ± SEM from three independent biological replicates. For RIP-qPCR in (**C**), TEDAR enrichment was calculated relative to the matched IgG control for each replicate, and statistical significance was assessed using a one-sample t-test against a hypothetical value of 1. For qRT-PCR in (**G**), relative expression of TEDAR and FLG was calculated after normalization to 18S and expressed relative to the siCtrl + DMSO condition. Statistical analysis was performed using one-way ANOVA followed by Tukey’s multiple-comparisons test on replicate-level values. *P < 0.05, **P < 0.01.

Because TEDAR localizes predominantly to the cytoplasm, we sought cytoplasmic effectors that could connect TEDAR to KLF4 localization control. RNA pull-down followed by mass spectrometry identified TEDAR-associated proteins enriched for the ERK1/2 signaling network (Fig. 6A, B and Supplementary Table S7), a known regulator of differentiation [26–28]. RNA immunoprecipitation (RIP) showed the enrichment of TEDAR in ERK1/2 immunoprecipitants from both keratinocyte and epidermal tissue lysates (Fig. 6C).

Since ERK activation has been linked to the cytoplasmic sequestration of KLF4 in other contexts [29, 30], we hypothesized that TEDAR promotes differentiation by suppressing ERK activity. Indeed, *TEDAR* knockdown led to a significant increase in phosphorylated ERK (p-ERK) levels without affecting total ERK protein (Fig. 6D). To test causality, i.e. whether ERK is involved in KLF4 regulation by TEDAR in keratinocytes, we treated TEDAR-depleted keratinocytes - which exhibit cytoplasmic KLF4 - with U0126, a MEK inhibitor, which reduced p-ERK levels (Fig. 6E). Notably, ERK inhibition successfully restored KLF4 nuclear localization in TEDAR-depleted cells (Fig. 6F) and rescued the expression of the terminal differentiation marker *FLG* (Fig. 6G). We also performed RNA secondary-structure prediction using RNAfold, which predicted multiple stem-loop regions within TEDAR, which is compatible with the possibility that TEDAR may provide a structured RNA platform for multivalent or indirect interactions with ERK, and/or its upstream regulators (Supplementary Fig. S8).

These data support a model in which *TEDAR* associates with the ERK1/2 complex to restrain ERK phosphorylation, thereby enabling KLF4 nuclear accumulation and the onset of terminal differentiation.

### TEDAR is reduced in inflammatory skin diseases and cSCC and is suppressed by IL-22/STAT3 signaling in epidermal models

**Fig. 7:**
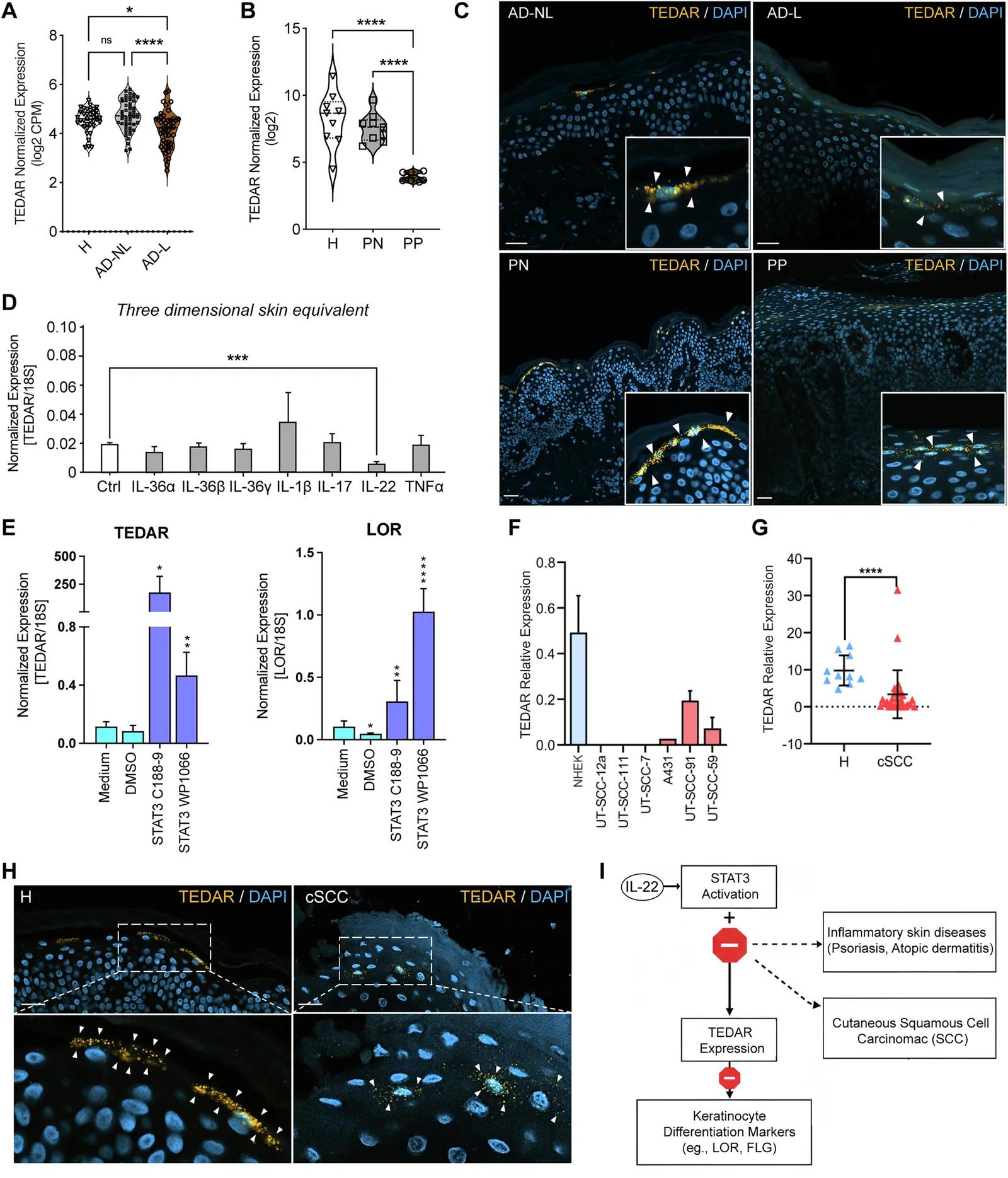
*TEDAR* is downregulated by cytokine signaling in cSCC and chronic inflammatory skin diseases with barrier perturbations. **A** Normalized TEDAR expression (log2 CPM) in healthy (H), atopic dermatitis non-lesional (AD-NL), and lesional (AD-L) skin (GSE121212). **B** Normalized TEDAR expression (log2) in healthy (H), psoriasis non-lesional (PN), and lesional (PP) skin (GSE274560). **C** RNAscope of TEDAR (yellow) in AD-NL, AD-L, PN, and PP tissue sections. Arrowheads indicate TEDAR signal; insets show higher magnification. Scale bars, 50 µm. **D** qRT-PCR of TEDAR in 3D epidermal equivalents treated with the indicated cytokines (*n* = 3). **E** qRT-PCR of TEDAR (left) and LOR (right) in primary keratinocytes treated with STAT3 inhibitors C188-9 or WP1066 (*n* = 3). **F** qRT-PCR of TEDAR in cSCC cell lines compared to NHEKs (*n* = 3). **G** qRT-PCR of TEDAR in primary cSCC tumors compared to healthy skin. **H** RNAscope of TEDAR (yellow) in healthy skin and cSCC tissue. Arrowheads indicate TEDAR signal in the granular layer of healthy epidermis; note the loss of signal in cSCC. Scale bars, 50 µm. **I** Schematic: IL-22–driven STAT3 activation suppresses TEDAR, linking inflammatory and oncogenic signaling to impaired keratinocyte terminal differentiation in psoriasis, atopic dermatitis, and cSCC. Data are mean ± SEM. *P < 0.05, **P < 0.01, ***P < 0.001, ****P < 0.0001 (Kruskal–Wallis with Dunn’s post-hoc test for (**A, B**); one-way ANOVA with Dunnett’s test for (**D, E**); Mann–Whitney U test for (**G**)).

Since our findings showed that *TEDAR* is needed for proper terminal differentiation, we hypothesized that its expression may be dysregulated in chronic inflammatory skin diseases characterized by barrier defects and epidermal dysfunction. Analysis of RNA-seq data from patients with atopic dermatitis (AD) and psoriasis revealed reduced *TEDAR* expression in lesional skin compared to non-lesional or healthy controls (Fig. 7A, B). In line with this, results from RNAscope analysis on patient samples revealed an aberrant and disorganized *TEDAR* expression in the granular layer of AD and psoriasis lesions (Fig. 7C).

To understand the mechanism behind this repression, we tested whether inflammatory cytokines implicated in these diseases regulate *TEDAR* expression. Treatment of 3D epidermal equivalents with a panel of cytokines (IL-36 isoforms, IL-1β, IL-17, IL-22, and TNFα) revealed that IL-22, a key driver of keratinocyte proliferation and inhibitor of differentiation, significantly suppressed *TEDAR* expression (Fig. 7D). Because IL-22 signals via STAT3, we treated keratinocytes with the STAT3 inhibitors (C188-9 or WP1066), which increased *TEDAR* levels and induced the differentiation marker *LOR* (Fig. 7E).

The IL-22/STAT3 signaling axis is not only central to skin inflammation but is also a driver of cutaneous squamous cell carcinoma (cSCC) [31–33], a malignancy characterized by impaired differentiation. Consistent with a STAT3-mediated repression mechanism, we found that *TEDAR* expression was significantly reduced in cSCC cell lines and primary tumors compared to healthy skin (Fig. 7F, G), and RNAscope analyses further confirmed the loss of *TEDAR* signal in cSCC tissue (Fig. 7H). Collectively, these results support a model in which pathogenic IL-22/STAT3 signaling suppresses *TEDAR*, linking inflammatory signaling to compromised terminal differentiation and barrier gene programs observed in both inflammatory skin diseases and cSCC (Fig. 7I).

## Discussion

We identify the lncRNA *TEDAR* as an important differentiation-stage-restricted regulator of terminal epidermal differentiation. *TEDAR* exhibits one of the most spatially restricted expression patterns in the human body, confined exclusively to the uppermost granular layer of the human epidermis. Importantly, *TEDAR* is both sufficient to induce late differentiation gene programs and required for late marker expression, keratohyalin granule abundance, and proper stratum corneum formation, identifying it as a novel, important element in the complex regulatory machinery leading to a functional skin barrier.

Our findings resolve the function of a transcript previously noted for its skin specificity [18–21]. We show that TEDAR expression is not merely skin-enriched, but strictly confined to the granular layer of keratinized epithelia, while being absent from non-keratinized stratified epithelium such as the buccal mucosa. The extraordinary spatial restriction of *TEDAR* expression, its absence in non-keratinizing mucosal epithelia, and its apparent absence in rodents raise the hypothesis that *TEDAR* may have evolved to meet the unique structural demands of the cornified envelope in certain lineages. However, we cannot exclude the existence of functionally analogous transcripts in other species, and the evolutionary origin and functional conservation of TEDAR will require dedicated comparative studies. Interestingly, this locus was originally annotated as a putative protein-coding gene, xp33, in the late 1990s [18]. However, our data support its reclassification as a functional non-coding RNA. We found no evidence of xp33 peptides either in public skin proteomics datasets [22] or in our own ORF expression assays, indicating that the transcript exerts its pro-differentiation effects as an RNA molecule. Nevertheless, as for any other lncRNAs, the possibility of a cryptic or dual coding/non-coding function cannot be completely ruled out and needs further investigation.

The broad impact of *TEDAR* on the cornification machinery suggests that it acts as an upstream regulator of the granular-to-cornified transition. Its depletion does not merely reduce a few markers but leads to a systemic collapse of the cornification machinery, including structural proteins (FLG, LOR), crosslinking enzymes (TGM1, TGM3), and lipid-processing enzymes (ALOX12B). While molecular details remain to be clarified, our data support a model where *TEDAR* may function as a cytoplasmic scaffold that physically interacts with ERK1/2 complex to permit the nuclear accumulation of KLF4, a zinc-finger transcription factor essential for barrier acquisition [24, 25]. These findings are in line with observations reported by the Mitchell group in embryonic stem cells, where nuclear localization of KLF4 has been shown to be dependent on ERK-activity [29]. This model could explain why *TEDAR* loss largely phenocopies the barrier defects seen in KLF4-deficient tissues, which is supported by our finding that ERK inhibition can restore KLF4 nuclear localization and rescue differentiation in TEDAR-depleted cells. Interestingly, there may exist a positive feedback loop between TEDAR and KLF4: knockdown of KLF4 resulted in decreased TEDAR induction upon CRISPR-activation, confirming results by Smirnov et al. [21] that TEDAR is directly or indirectly under the regulation of KLF4. The finding that TEDAR regulates ERK1/2-phosphorylation adds *TEDAR* to a growing list of lncRNAs that modulate post-translational modifications of signaling proteins [34–36]. However, it remains to be determined whether TEDAR interacts with ERK1/2 directly or indirectly through bridging proteins.

Our findings showed downregulation of *TEDAR* in cSCC and its disorganized expression in atopic dermatitis and psoriasis, conditions characterized by barrier dysfunction and disturbed differentiation. Suppression of *TEDAR* by the IL-22/STAT3 axis establishes a link between inflammation and differentiation abnormalities, with relevance to chronic inflammatory skin diseases as well as keratinocyte cancer. Biologically, IL-22 inhibits terminal differentiation and enhances antimicrobial defence and proliferation in epithelia, a response protective during acute injury but maladaptive during chronic inflammation [37–39]. Our findings suggest that the IL-22-mediated repression of *TEDAR* could be a link between inflammation and impaired differentiation in psoriasis and AD. Similarly, in cSCC, which is also linked to the overexpression of IL-22 and its receptor (IL-22R1) [31–33], the loss of *TEDAR* may contribute to the undifferentiated and invasive phenotype of tumor cells as well as maintenance of stemness. Therefore, restoring *TEDAR* expression or mimicking its function could represent a potential therapeutic strategy to restore differentiation and barrier integrity in these conditions. A limitation of our study is that the disease associations described here are based on correlative expression analyses; the direct functional contribution of TEDAR loss to the pathogenesis of psoriasis, AD, or cSCC, as well as its potential benefit for therapeutic restoration of TEDAR, will require functional validation in disease models.

Finally, *TEDAR* shows strong conservation among primates but limited conservation in rodents, highlighting a potential human/primate-specific layer of barrier regulation, although we cannot exclude the existence of functional analogs in other species and the identification of putative TEDAR-orthologues, as well as their functionality, is warranted. This possibility may help explain why certain aspects of late epidermal differentiation and inflammatory barrier disease are incompletely modeled in mice or other rodents and underscores the value of human tissue and organotypic systems for dissecting non-coding regulators of skin biology. The genomic localization of *TEDAR*, residing within the late cornified envelope (*LCE*) gene cluster, flanked by *LCE3A* and *LCE2D*, together with its partial sequence similarity to *LCE* genes, raise the possibility that it may have arisen during evolution through pseudogenization of a protein-coding ancestor, a mechanism described for lncRNA genes (for example *XIST* [40]), and that it was thereafter preserved due to a newly acquired function.

In summary, *TEDAR* is a primate-conserved lncRNA confined to the granular layer of cornified epithelia that drives terminal epidermal differentiation by restraining ERK phosphorylation, thereby enabling KLF4 nuclear accumulation. Its repression by IL-22/STAT3 signaling provides a mechanistic link between inflammation and barrier failure in common skin diseases. These findings provide new insights into the genetic networks governing human skin biology and highlight the importance of non-coding RNAs in maintaining tissue homeostasis.

## Supplementary information

Supplementary Information

Supplementary Figure 1

Supplementary Figure 2

Supplementary Figure 3

Supplementary Figure 4

Supplementary Figure 5

Supplementary Figure 6

Supplementary Figure 7

Supplementary Figure 8

Supplementary Table 1.

Supplementary Table 2.

Supplementary Table 3.

Supplementary Table 4.

Supplementary Table 5.

Supplementary Table 6.

Supplementary Table 7.

Supplementary Table 8.

Supplementary Table 9.

Full-length uncropped original Western blots

## Data Availability

Transcriptomic data generated in this study have been deposited in the NCBI Gene Expression Omnibus (GEO) under accession numbers GSE302073 and GSE302049. Publicly available datasets analyzed in this study are available under accessions GSE121212 (Atopic Dermatitis) and GSE274560 (Psoriasis).
