# Supplementary material for: The skin-specific long non-coding RNA TEDAR orchestrates late epidermal differentiation via an ERK-KLF4 cytoplasmic regulatory axis
Source: Cell Death Dis. 2026 Sep 5;17(1):779. doi: 10.1038/s41419-026-09192-0 (PMC13550425; doi:10.1038/s41419-026-09192-0)
Supplement: Supplementary file 1 [file 41419_2026_9192_MOESM19_ESM.pdf]

Suppl. 3a 5' and 3' RACE for TEDAR

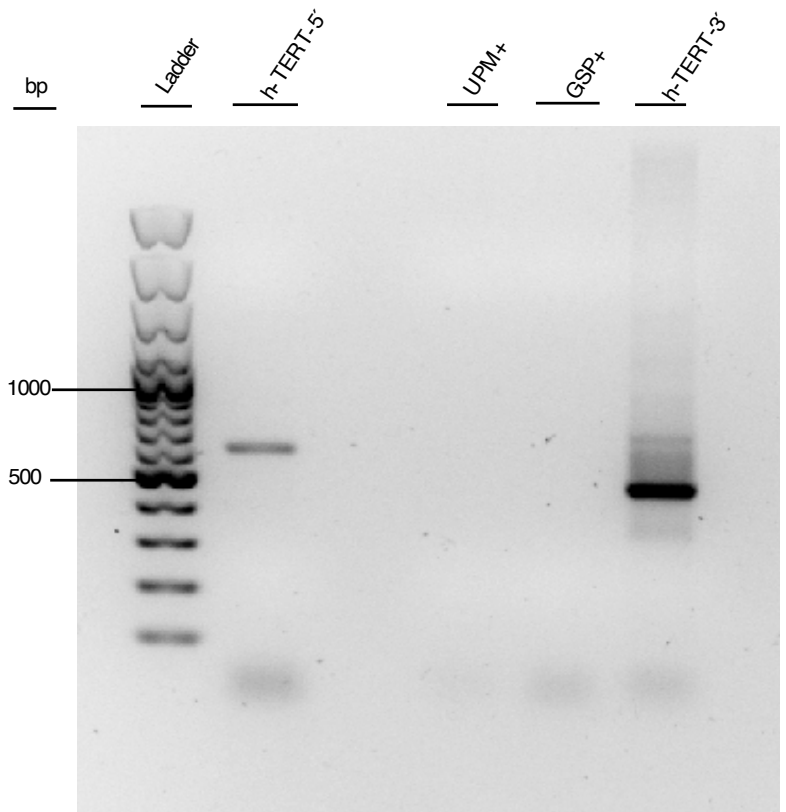

5' RACE product (approx 650 bp)
3' RACE product (approx 450 bp)

UPM- Universal Primer Mix
GSP- Gene specific Primer

02/06/2021

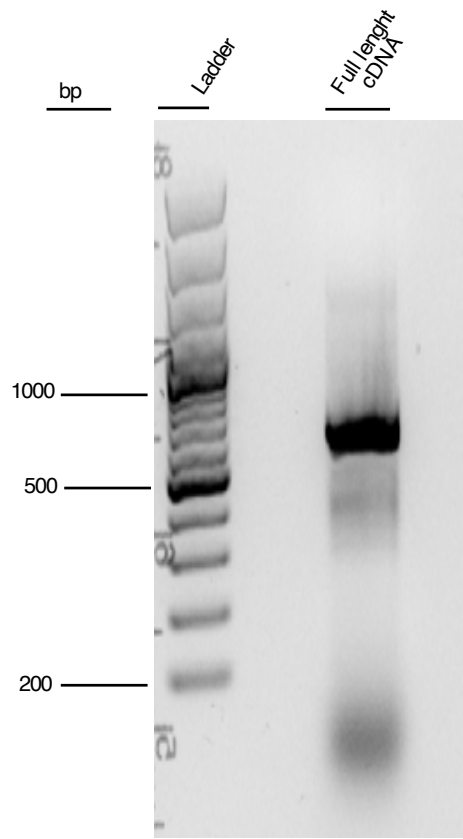

• Full length transcript is 589 nt

02/06/2021

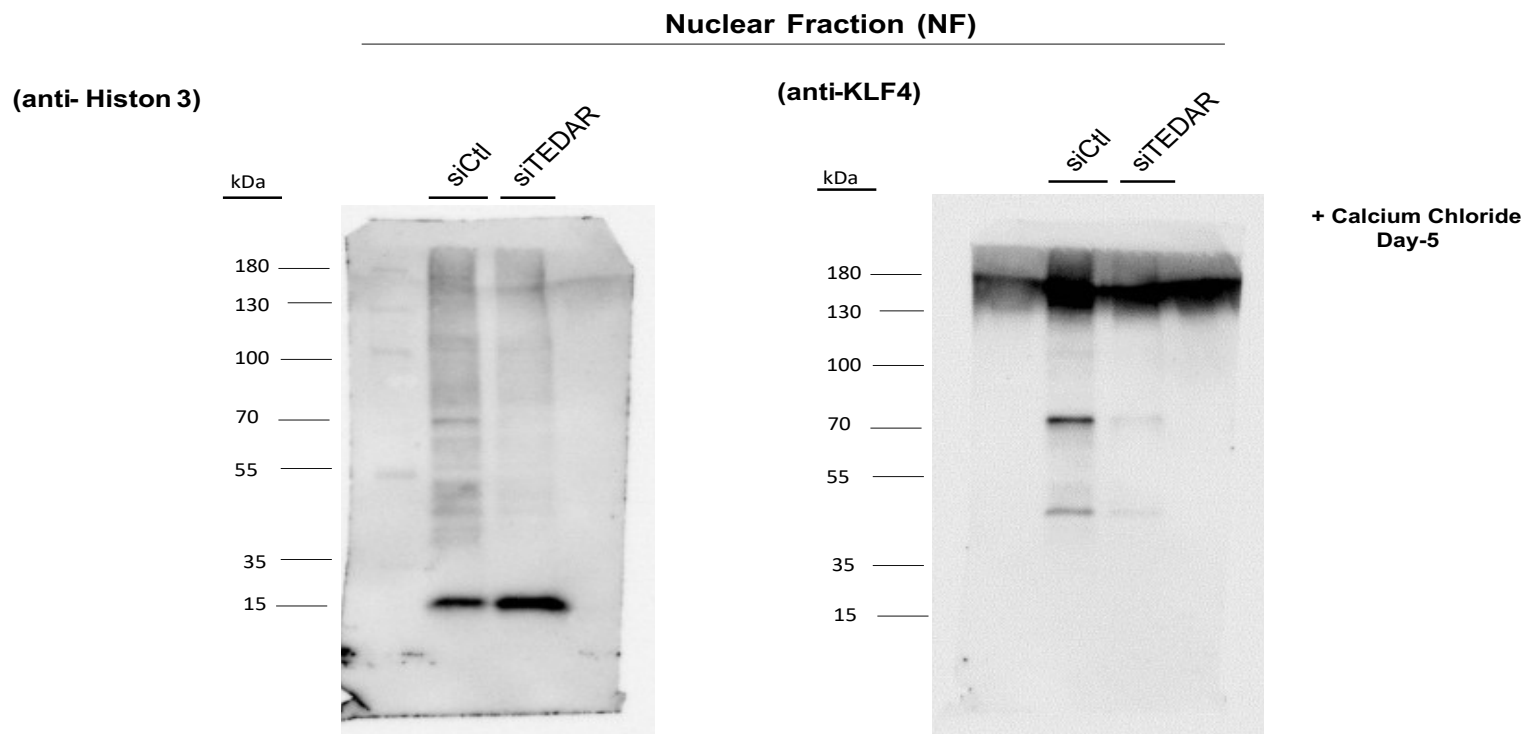
Nuclear Fraction (NF)
(anti- Histon 3)
kDa
siCtl
siTEDAR
180
130
100
70
55
35
15
(anti-KLF4)
kDa
siCtl
siTEDAR
180
130
100
70
55
35
15
+ Calcium Chloride
Day-5

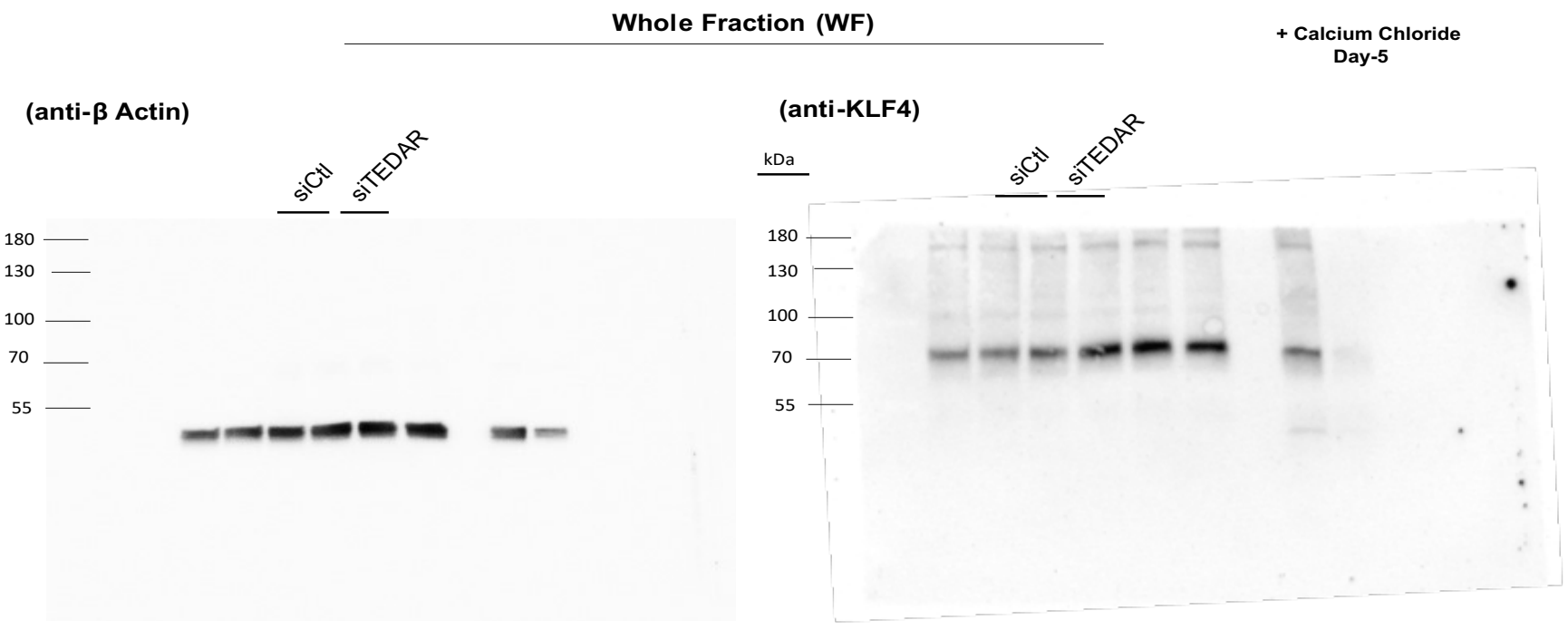
Whole Fraction (WF)
+ Calcium Chloride
Day-5
(anti-β Actin)
siCtl
siTEDAR
180
130
100
70
55
(anti-KLF4)
kDa
siCtl
siTEDAR
180
130
100
70
55

Figure 6b RNA-immunoprecipitation experiment results of Ca2+-treated (day 5) N/TERT cells

Agarose gel

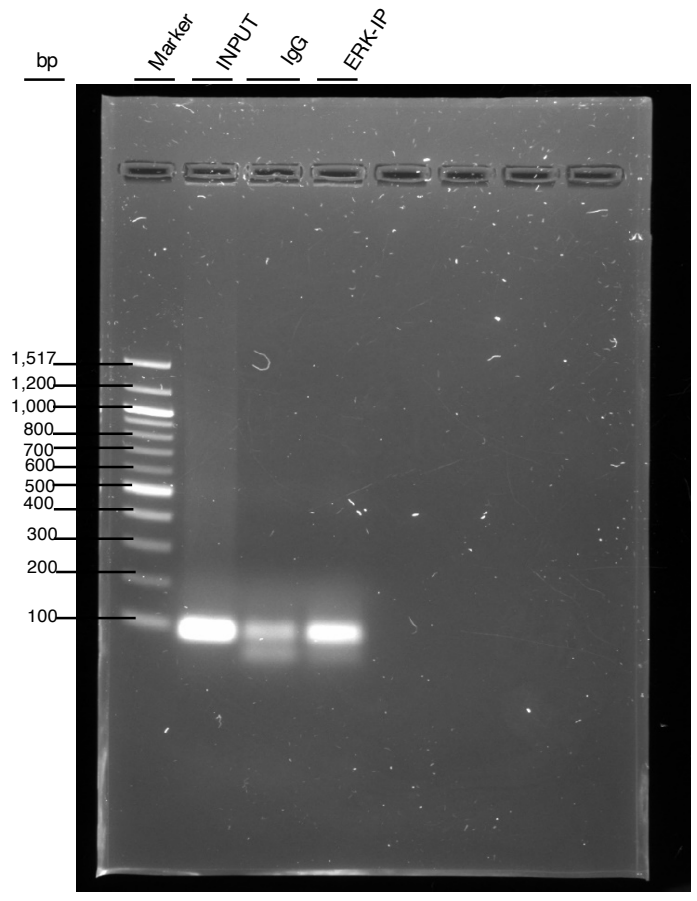

Primer seq

Forward: GAGCACAACTGAAAGGGTTGACT
Reverse: GTAGTGGGCCCTTTAAGAGGA

Product size: 93 nt

15/09/2023

WB

(anti - ERK)

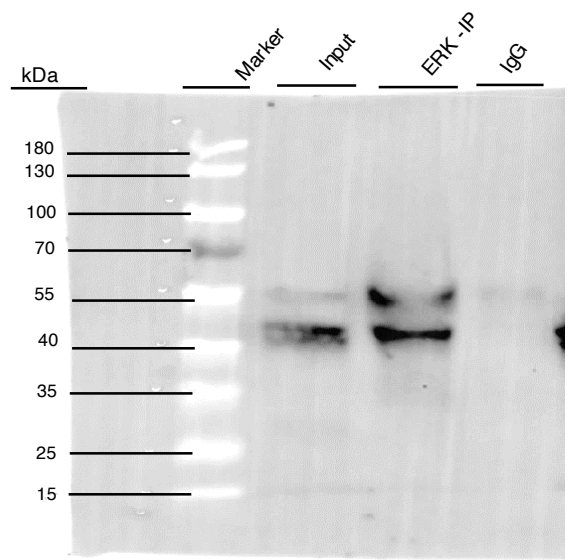

11/10/2023

Figure 6c RNA-immunoprecipitation experiment results of Ca2+-treated (day 5) N/TERT cells

Agarose gel

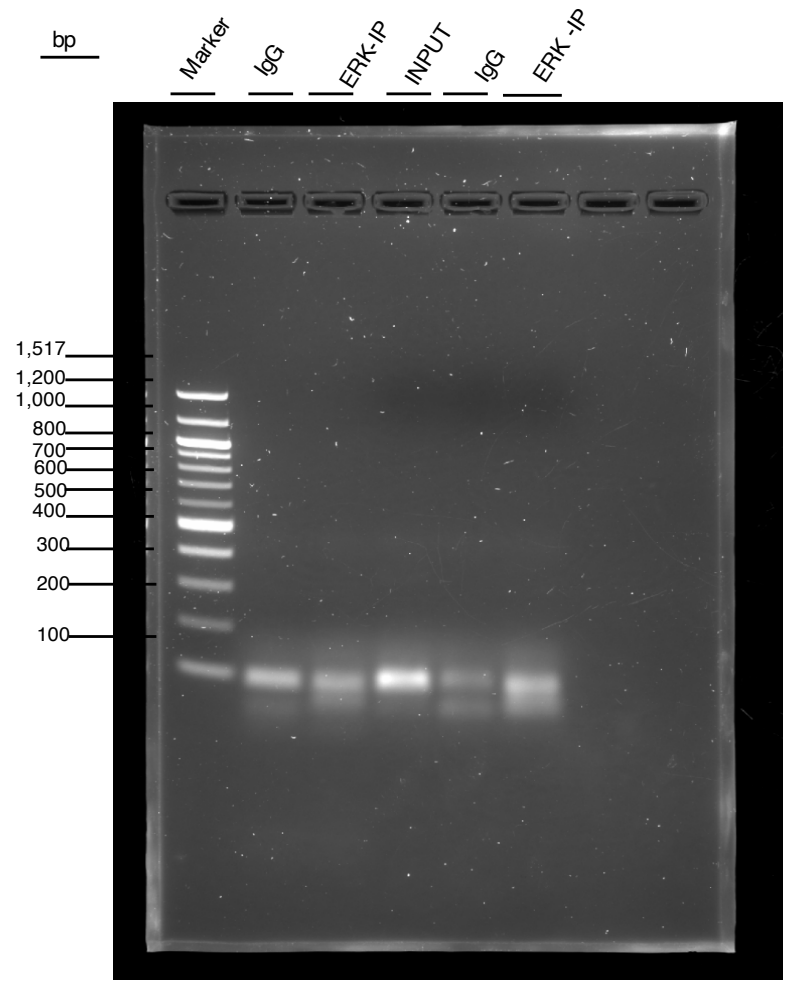

Primer seq

Forward: GAGCACAACTGAAAGGGTTGACT
Reverse: GTAGTGGGCCCTTTAAGAGGA

Product size: 93 nt

02/06/2023

WB

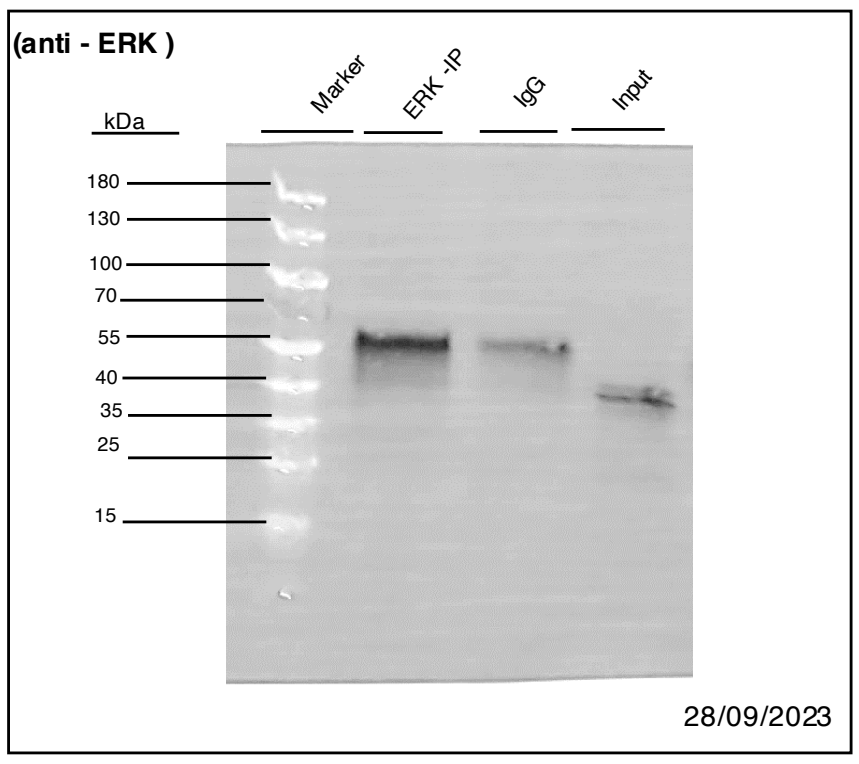

Phosphorylation status of ERK protein after TEDAR downregulation and effect of U0216 inhibitor followed by Ca2+ treatment in N/HEK cells

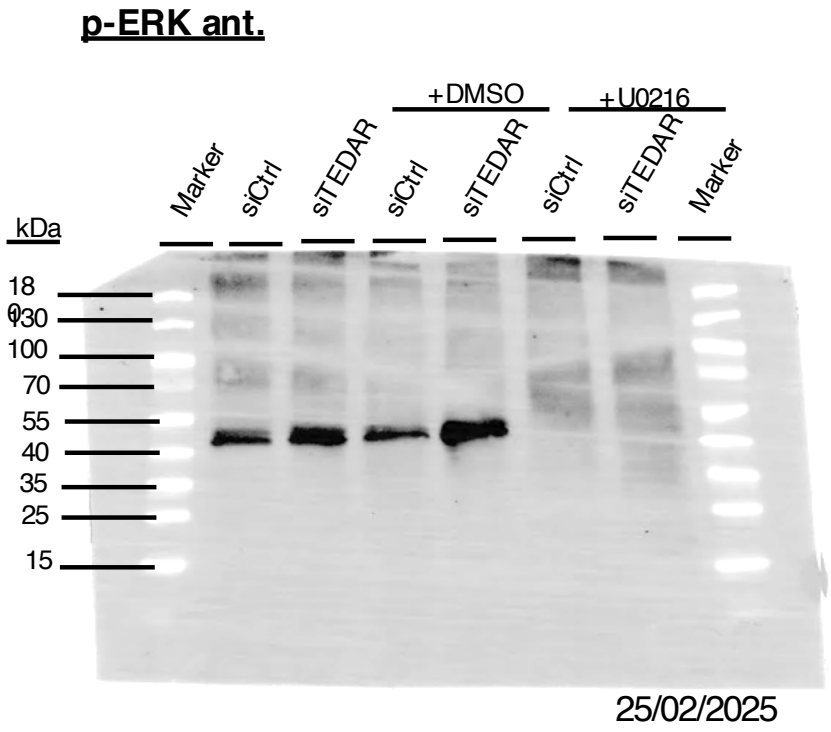

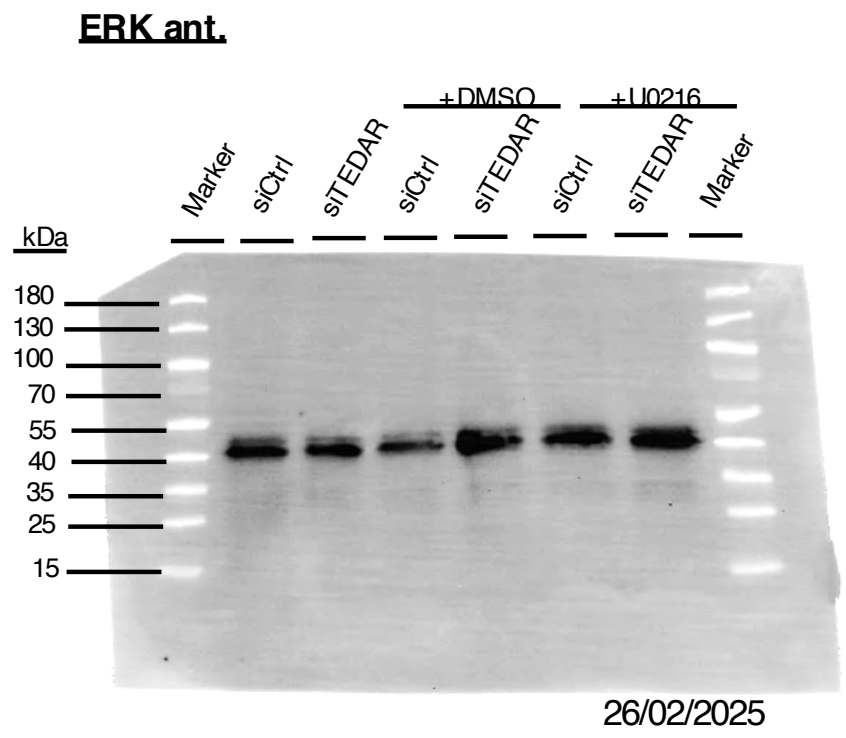

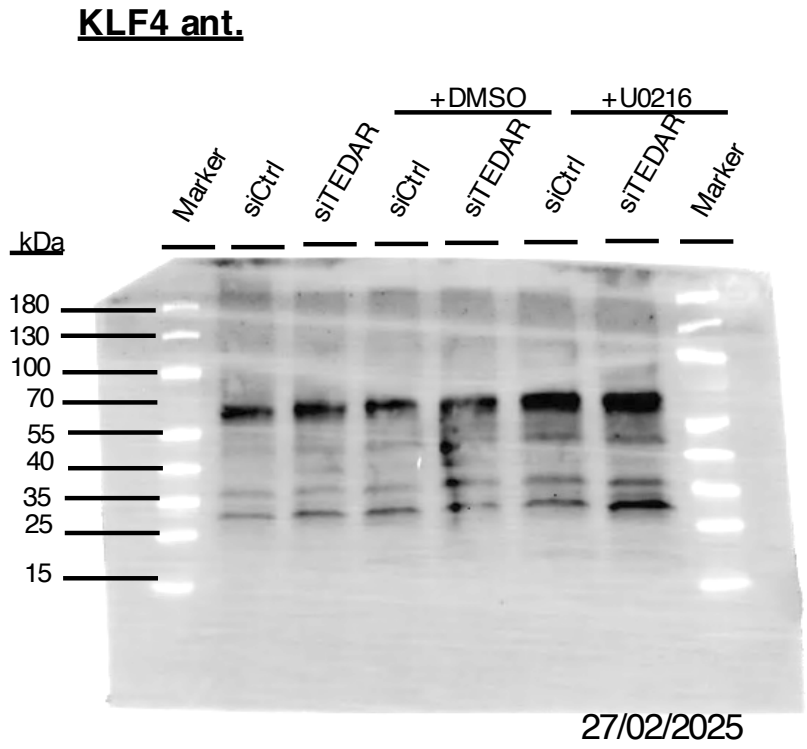

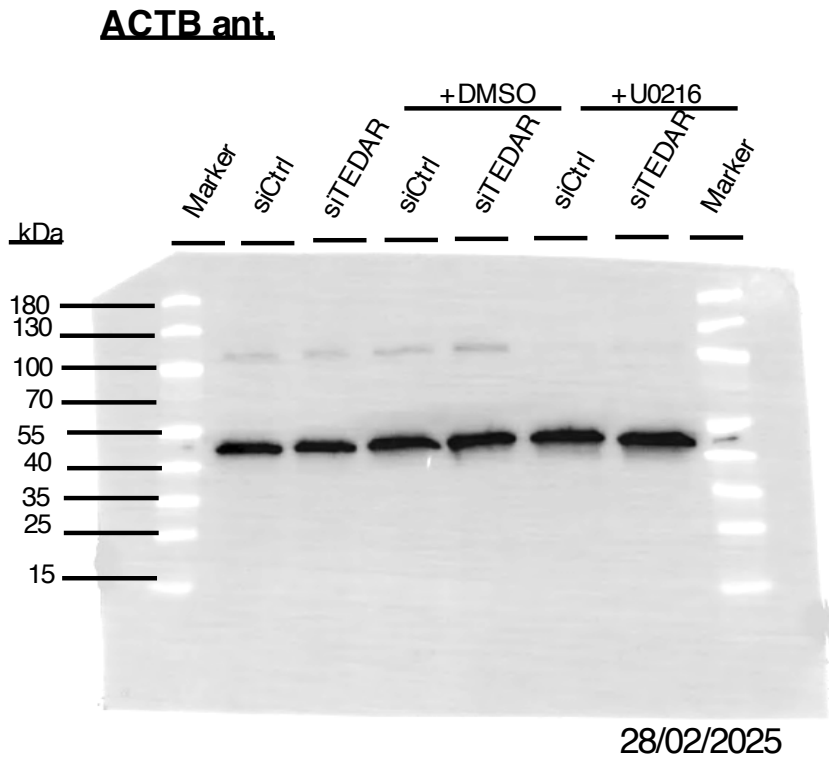

*** Stripping was done and ACTB was used as a loading control.**
