# Supplementary material for: The skin-specific long non-coding RNA TEDAR orchestrates late epidermal differentiation via an ERK-KLF4 cytoplasmic regulatory axis
Source: Cell Death Dis. 2026 Sep 5;17(1):779. doi: 10.1038/s41419-026-09192-0 (PMC13550425; doi:10.1038/s41419-026-09192-0)
Supplement: Supplementary file 2 [file 41419_2026_9192_MOESM1_ESM.docx]

**Supplementary Information**

**1. Supplementary Methods**

**Clinical Samples**

Skin biopsies (4 mm) were obtained from healthy donors and patients with atopic dermatitis, psoriasis, cutaneous squamous cell carcinoma (cSCC) at the Karolinska University Hospital, Stockholm, Sweden, after written informed consent. Diagnosis was confirmed by histopathology. Formalin-fixed, paraffin-embedded (FFPE) tissue from healthy skin, buccal mucosa, and cSCC was obtained from the Karolinska University Hospital Biobank, while buccal mucosa samples were obtained from the Odontology Biobank. The study was approved by the Swedish Ethical Review Authority and conducted according to the Declaration of Helsinki.

**Cell culture and treatments**

Primary NHEKs were obtained from Thermo Fisher Scientific and used at low passage according to the supplier’s recommendations. N/TERT2G keratinocytes generated in the J. Rheinwald laboratory (Harvard Medical School, Boston, USA)^1,2^ were obtained through collaboration and were cultured in EpiLife medium supplemented with Human Keratinocyte Growth Supplement (HKGS). Differentiation was induced by adding 1.5 mM CaCl2. cSCC cell lines (UT-SCC-12a, -111, -7, -91, -59, and A431) were cultured in DMEM with 10% FBS. For cytokine treatments, cells or 3D tissues were incubated with recombinant human IL-22, IL-17A, IL-1β, IL-36α, IL-36β, IL-36γ, or TNFα (R&D Systems; 20–100 ng/mL) for 24–48 h. For STAT3 inhibition, cells were treated with C188-9 (10 µM) or WP1066 (5 µM; Selleckchem) for 24 h.

**CRISPR-Mediated Transcriptional Activation**

The CRISPR/Cas9 Synergistic Activation Mediator (SAM) system comprising three plasmids (the sgRNA (MS2) cloning backbone (Plasmid #61424), the MS2-P65-HSF1_GFP (Plasmid #61423), and the dCAS9-VP64_GFP (Plasmid #61422)) was purchased from Addgene (MA, USA). Four sgRNAs (designated as sgRNA1-4) targeting positions −500 to +1 relative to the *TEDAR* transcriptional start site (TSS) were designed using the MIT CRISPR Design Tool (crispr.mit.edu); no high-confidence off-target sites with fewer than two mismatches were identified in the human genome (Supplementary Fig. S3A-B). The sgRNA expression plasmids were constructed by annealing complementary oligonucleotide pairs and ligating them into the BbsI-linearized sgRNA (MS2) cloning backbone according to the SAM cloning protocol¹ (<https://www.addgene.org/crispr/zhang/>). Correct insertion was verified by Sanger sequencing. Only sgRNA2 could induce *TEDAR* expression robustly and was therefore used in all subsequent experiments. The sequences of sgRNA2 oligos used for cloning were: Forward: 5′-CACCGGCCTTAGTGAGCCATGGTTC-3′; Reverse: 5′-AAACGAACCATGGCTCACTAAGGCC-3′. Primary adult keratinocytes (passage 3) at 60–70% confluence were transfected with the sgRNA (MS2), MS2-P65-HSF1_GFP, and dCAS9-VP64-GFP plasmids using Lipofectamine™ 2000 (Thermo Fisher Scientific).

**RNA interference**

For RNA interference, 5 × 10⁶ NHEKs were electroporated with 1 nmol siPools targeting *TEDAR* or non-targeting controls (siTOOLs Biotech) using the Amaxa Nucleofector (Lonza; program T-018). For CRISPRa, cells were transfected with sgRNA (MS2) cloning backbone (Addgene #61424), MS2-P65-HSF1_GFP (#61423), and dCAS9-VP64_GFP (#61422) using Lipofectamine 2000 (Thermo Fisher Scientific). For combined TEDAR activation and KLF4 depletion, N/TERT2G keratinocytes were transfected with the SAM components and TEDAR-targeting sgRNA2 or non-targeting control sgRNA as described above. After medium replacement, cells were transfected with siRNA targeting KLF4 or non-targeting control siRNA and subsequently induced to differentiate with high-calcium medium. Cells were harvested on day 6 for qRT-PCR analysis of TEDAR, KLF4 and late differentiation markers. Three independent biological replicates were analyzed.

**3D organotypic epidermal equivalents**

3D skin models were generated as described previously ^3^. Briefly, 1.75 × 10⁵ keratinocytes were seeded onto polycarbonate inserts (Thermo Fisher) in EpiLife with 1.5 mM CaCl2 for 48 h. Cultures were then air-lifted and maintained in differentiation medium supplemented with 50 µg/mL ascorbic acid and 10 ng/mL KGF. Models were harvested at day 4 post-airlifting for analysis.

**Subcellular fractionation**

Differentiated keratinocytes were lysed in cytoplasmic lysis buffer (0.15% NP-40, 10 mM Tris-HCl pH 7.0, 150 mM NaCl) and layered onto a sucrose cushion (25% sucrose) for centrifugation (13,000 rpm, 10 min, 4°C). The supernatant (cytoplasm) was collected, and the nuclear pellet was washed three times in nuclei wash buffer (0.1% Triton-X-100) before lysis in high-salt buffer (1% NP-40, 300 mM NaCl, 1M Urea). Fraction purity was verified by Western blot.

**Gene expression analysis**

Total RNA was isolated using TRIzol (Invitrogen) or RNeasy Plus Mini Kit (Qiagen). cDNA was synthesized using the RevertAid First Strand Synthesis Kit (Thermo Fisher). qRT-PCR was performed using SYBR Green assays (Thermo Fisher) on a QuantStudio 7 system. Expression was normalized to *18S* rRNA using the ΔCt method. 5′ and 3′ RACE were performed using the SMARTer RACE Kit (Takara Bio) on RNA from differentiated keratinocytes. Primers are listed in **Supplementary Table S8**.

**Western blotting**

Lysates were prepared in RIPA buffer supplemented with protease inhibitors (Roche). Proteins (15 µg) were resolved by SDS-PAGE, transferred to nitrocellulose membranes, and incubated with primary antibodies overnight at 4°C. Detection was performed using HRP-conjugated secondary antibodies and SuperSignal West Femto substrate (Life Technologies). Antibodies are listed in **Supplementary Table S9**.

**Histology, Immunofluorescence, and RNAscope**

For immunofluorescence, 7 µm cryosections or fixed cells were blocked in 10% BSA and incubated with primary antibodies overnight, followed by Alexa Fluor-conjugated secondary antibodies. Nuclei were stained with Hoechst 33342. Single-molecule RNA *in situ* hybridization was performed using RNAscope Multiplex Fluorescent Reagent Kit v2 (ACD) with probes targeting *TEDAR*. Images were acquired on a Zeiss LSM 900 Airy2 confocal microscope.

**RNA immunoprecipitation (RIP) and Pull-down**

For RIP, cross-linked cells or tissues were lysed, sonicated, and incubated with anti-ERK1/2 or IgG control antibodies overnight. Immunocomplexes were captured on Protein G beads, and RNA was extracted for qRT-PCR. For RNA pull-down, biotinylated full-length *TEDAR* or antisense RNA was transcribed *in vitro* (MEGAscript T7 Kit, Thermo Fisher) and incubated with cell lysates. Complexes were captured on streptavidin beads, eluted, and analyzed by mass spectrometry.

**Transcriptomic and Bioinformatics analysis**

To systematically identify skin-enriched transcripts, particularly long non-coding RNAs (lncRNAs), we analyzed publicly available RNA-sequencing data from the Genotype-Tissue Expression (GTEx) v8 project. The data used for the analyses described in this manuscript were obtained from the GTEx Portal (<https://gtexportal.org/> ). To focus the analysis on physiological solid-tissue transcriptomes, confounding samples derived from transformed cell lines (e.g., EBV-transformed lymphocytes, cultured fibroblasts) and highly variable mucosal sites, as well as testicular tissue, were excluded prior to analysis. Raw RNA-seq counts were processed using the Seurat (v4.0) workflow. To mitigate technical variation, samples were normalized and variance-stabilized using SCTransform, with the RNA Integrity Number (RIN) regressed as a latent variable. Following dimensionality reduction (t-SNE/UMAP), clusters were annotated based on tissue-of-origin metadata. Differential expression analysis was then performed to identify transcripts exhibiting highly restricted expression in the skin. Finally, Ensembl biomaRt annotations were applied to systematically exclude protein-coding genes and isolate the final signature of skin-enriched long non-coding RNAs (lncRNAs).

For disease association analyses, RNA-seq datasets for Atopic Dermatitis (GSE121212) and Psoriasis (GSE274560) were retrieved from NCBI Gene Expression Omnibus (GEO) and TEDAR expression was compared across conditions. 3D organotypic models were profiled using Affymetrix HuGene-2.1 arrays. Differential expression analyses upon CRISPR-activation or knockdown were performed and heatmaps were generated in R using the pheatmap package. Downstream pathway analyses were performed using Gene Set Enrichment Analysis (GSEA)^4^, Reactome^5^ and Enrichr^6^. RNA secondary structure prediction was performed using RNAfold from the ViennaRNA Package, based on minimum free energy (MFE) calculations and thermodynamic parameters^7–9^.

**Statistical analysis**

Experiments were performed using independent biological replicates as indicated in the figure legends. For cell-culture experiments, biological replicates represent independently cultured and treated cell preparations; for 3D organotypic models, biological replicates represent independently generated epidermal equivalents; and for clinical samples, biological replicates represent independent donor or patient samples. No formal statistical power calculation was performed; sample sizes were selected based on prior experience with comparable keratinocyte, organotypic skin model, imaging and transcriptomic analyses. No samples were excluded from analysis except for technical failures, such as insufficient RNA quality, failed amplification, insufficient tissue integrity, or failed staining/imaging quality. No formal randomization procedure was used; parallel cultures were allocated to treatment or control conditions within each experiment and processed identically. Investigators were not blinded during routine cell-culture, qRT-PCR, western blot or transcriptomic analyses; where applicable, image quantification was performed using identical acquisition and analysis settings across groups. For small-n biological replicate experiments, formal normality and equal-variance testing was not considered reliable; therefore, statistical tests were selected based on experimental design and data type, with non-parametric tests used where appropriate. Statistical tests were two-sided unless otherwise stated, and multiple-comparison corrections were applied as indicated in the figure legends. Data are shown as mean ± SEM unless otherwise stated, and P < 0.05 was considered statistically significant.

**Code availability**

Bioinformatic analyses were performed using R and publicly available software/packages as described above, including Seurat v4.0, biomaRt, pheatmap, GSEA, Reactome, Enrichr and RNAfold from the ViennaRNA Package. No custom software was developed for this study. Analysis scripts used to generate the transcriptomic and enrichment analyses are available from the corresponding author upon reasonable request.

**References**

1	Dickson MA, Hahn WC, Ino Y, Ronfard V, Wu JY, Weinberg RA *et al.* Human keratinocytes that express hTERT and also bypass a p16(INK4a)-enforced mechanism that limits life span become immortal yet retain normal growth and differentiation characteristics. *Mol Cell Biol* 2000; **20**: 1436–1447.

2	Smits JPH, Niehues H, Rikken G, van Vlijmen-Willems IMJJ, van de Zande GWHJF, Zeeuwen PLJM *et al.* Immortalized N/TERT keratinocytes as an alternative cell source in 3D human epidermal models. *Sci Rep* 2017; **7**: 11838.

3	Luo L, Pasquali L, Srivastava A, Freisenhausen JC, Pivarcsi A, Sonkoly E. The Long Noncoding RNA LINC00958 Is Induced in Psoriasis Epidermis and Modulates Epidermal Proliferation. *J Invest Dermatol* 2023; **143**: 999–1010.

4	Subramanian A, Tamayo P, Mootha VK, Mukherjee S, Ebert BL, Gillette MA *et al.* Gene set enrichment analysis: a knowledge-based approach for interpreting genome-wide expression profiles. *Proc Natl Acad Sci U S A* 2005; **102**: 15545–15550.

5	Ragueneau E, Gong C, Sinquin P, Sevilla C, Beavers D, Grentner A *et al.* The Reactome Knowledgebase 2026. *Nucleic Acids Res* 2026; **54**: D673–D681.

6	Xie Z, Bailey A, Kuleshov MV, Clarke DJB, Evangelista JE, Jenkins SL *et al.* Gene Set Knowledge Discovery with Enrichr. *Curr Protoc* 2021; **1**: e90.

7	Mathews DH, Disney MD, Childs JL, Schroeder SJ, Zuker M, Turner DH. Incorporating chemical modification constraints into a dynamic programming algorithm for prediction of RNA secondary structure. *Proc Natl Acad Sci U S A* 2004; **101**: 7287–7292.

8	Gruber AR, Lorenz R, Bernhart SH, Neuböck R, Hofacker IL. The Vienna RNA websuite. *Nucleic Acids Res* 2008; **36**: W70-74.

9	Lorenz R, Bernhart SH, Höner Zu Siederdissen C, Tafer H, Flamm C, Stadler PF *et al.* ViennaRNA Package 2.0. *Algorithms Mol Biol AMB* 2011; **6**: 26.

**2. Supplementary Figure Legend**

**Supplementary Figure 1. Characterization of skin-enriched RNAs and tissue-specific expression of *TEDAR*. A** Heatmap showing the top 500 skin-enriched mRNAs identified from the GTEx dataset. **B** Gene Ontology (GO) enrichment analysis of skin-enriched mRNAs (Biological Processes). **C** Violin plot comparing the abundance (log2 median TPM in skin) of skin-enriched non-coding RNAs and protein-coding mRNAs. *TEDAR* (*ENSG00000176075.7*) is highlighted in red. **D** Feature plots of *TEDAR* and *LOR* expression across human tissues (t-SNE). **E** qRT-PCR of *TEDAR* in 16 skin-resident cell types (n = 3). NHEK, normal human epidermal keratinocytes. **F** Representative RNAscope images of *TEDAR* (yellow) in healthy epidermis vs. negative control probe (Neg-Ctl) (n = 6). **G** RNAscope of *TEDAR* (yellow) in skin from multiple anatomical sites (arm, scalp, foot, back). Nuclei are counterstained with DAPI (blue). Scale bars, 50 µm. **H** RNA-seq signal tracks (GTEx) showing *TEDAR* expression in skin/NHEK vs. non-keratinizing mucosa (vagina, esophagus, bladder). Data are mean ± SEM.

**Supplementary Figure 2. Genomic location, expression kinetics, and conservation of *TEDAR*.** **A** Top: Map of the Epidermal Differentiation Complex (EDC) on chr1q21.3 showing *TEDAR* location. Bottom: RNA-seq signal tracks of *TEDAR*, *KRT10*, *FLG*, and *LOR* during keratinocyte differentiation (days 0–7) (GSE59827). **B** qRT-PCR of *TEDAR* and differentiation markers in cultured keratinocytes over a 6-day differentiation time course (n = 3). **C** qRT-PCR of *TEDAR* and *LCE2D* during 3D epidermal equivalent stratification (n = 3). **D** UCSC Genome Browser view showing conservation of the *TEDAR* locus (PhastCons). Red boxes indicate exons. Data are represented as mean ± SEM. Statistical significance was determined using Student’s *t* test (two-tailed). **P* < 0.05, ***P* < 0.01, ****P* < 0.001, *****P* < 0.0001.

**Supplementary Figure 3. sgRNA screening for CRISPRa-mediated TEDAR activation and transcriptional effects in proliferating and differentiating keratinocytes. A** Schematic of the TEDAR gene locus showing the positions of four sgRNAs (sg1–sg4) relative to the transcriptional start site (TSS, +1) and the TEDAR gene structure (blue box, exon; arrows, transcriptional direction). sgRNAs were tiled across the proximal promoter region (−500 to +1). **B** qRT-PCR of TEDAR expression following CRISPRa with each individual sgRNA (sgRNA 1–4) or non-targeting control (sg-Ctrl) in NHEKs (n = 3). sgRNA 2 was the only guide to robustly induce TEDAR expression and was selected for all subsequent experiments. **C** qRT-PCR analysis of early (*KRT10*) and late (*FLG*, *LOR*, *LCE2D*, *LCE3A*) differentiation genes in NHEKs following CRISPR-SAM-mediated *TEDAR* activation (sgTEDAR) vs. control (sgCtrl), cultured without calcium (n = 3). **D** qRT-PCR of differentiation markers (*KRT10*, *FLG*, *LOR*, *LCE2D* and *LCE3A*) in control (sgCtrl) vs. *TEDAR*-activated (sgTEDAR) cells with 4 days calcium-induced differentiation (n = 3). **E** Dot plot of enriched GO Biological Process terms among TEDAR-upregulated genes. Dot size indicates gene count; color indicates adjusted P value. **F** Gene Set Volcano plot of GO Biological Process terms showing normalized enrichment scores (NES) plotted against –log₁₀(adjusted p-value). Significantly enriched terms are highlighted in red. Data are mean ± SEM. Statistical significance was determined using Student’s *t* test (two-tailed). **P* < 0.05, ***P* < 0.01; ns, not significant.

**Supplementary Figure 4. *TEDAR* depletion does not affect epidermal thickness, proliferation or viability.** **A** Immunofluorescence of Keratin 1 (KRT1, red) and Collagen VII (Col VII, green) in siControl vs. siTEDAR 3D skin cultures. Scale bars, 50 µm. **B** Quantification of epidermal thickness (n = 3). **C** Left: Immunofluorescence of proliferation marker Ki-67 (magenta). Scale bars, 100 µm. Right: Quantification of Ki-67+ nuclei (n = 3). **D** Cell viability (PrestoBlue assay) in primary keratinocytes 24 h post-transfection (n = 3). **E** Caspase-3/7 activity assay (n = 3). Data are median with quartiles (**B**, **C**) or mean ± SEM (**D**, **E**). ns, not significant (Student’s t-test).

**Supplementary Figure 5. *TEDAR* depletion alters gene networks associated with cornification and upper epidermal differentiation. A** Top 10 enriched GO Biological Process terms for *TEDAR*-regulated genes. **B** GSEA volcano plot for GO Cellular Component terms. **C** GSEA enrichment plots (top) and heatmaps (bottom) for "Upper Epidermis" [GSE230152] and "Cornification" [Human Protein Atlas] gene sets. **D** Box plots of top downregulated barrier genes. **E** qRT-PCR validation of *CDSN* expression in 3D skin cultures (n = 3). Data are represented as mean ± SEM. Statistical significance was determined using Student’s *t* test (two-tailed). ****P* < 0.001.

**Supplementary Figure 6. Transcriptional convergence of *TEDAR* activation and depletion models.** **A** and **B** PCA plots (left) and hierarchical clustering heatmaps (right) of transcriptomic profiling data from **A** *TEDAR* activation (CRISPRa; n = 4) and **B** *TEDAR* knockdown (siRNA; n = 3). **C** Venn diagram showing overlap of differentially expressed genes (DEGs) between CRISPRa and siRNA datasets. **D** Reactome pathway analysis of the 118 commonly regulated genes. **E** Reactome pathway hierarchy (Voronoi treemap) showing enrichment of the "Developmental Biology" and "Cornified Envelope" modules.

**Supplementary Figure 7. KLF4 is required for TEDAR-mediated induction of epidermal differentiation genes. (A)** Experimental design for CRISPRa-mediated TEDAR activation and siRNA-mediated KLF4 knockdown during calcium-induced keratinocyte differentiation. N/TERT2G keratinocytes were subjected to TEDAR CRISPR activation, followed by siKLF4 transfection and high-calcium differentiation. Cells were harvested on day 6 for RNA analysis. **(B)** qRT-PCR analysis of **LCE1B** expression in control and TEDAR-activated keratinocytes with or without KLF4 knockdown. Data are presented as mean ± SEM from three independent biological replicates. Statistical significance was determined by one-way ANOVA with Tukey's multiple-comparisons test. ns, not significant; **P < 0.01; ****P < 0.0001.

**Supplementary Figure 8. Predicted secondary structure of lncRNA TEDAR.** RNA secondary structure prediction was performed using RNAfold from the ViennaRNA Package, based on minimum free energy (MFE) calculations and thermodynamic parameters. (see supplementary methods for further details). The structure exhibits multiple stem-loop regions and modular domains, consistent with a potential scaffold function.

**3. Supplementary Table Legends**

**Supplementary Table 1**. List of top skin-enriched mRNAs identified in this study (Excel file).

**Supplementary Table 2**. List of top skin-enriched non-coding transcripts identified in this study (Excel file).

**Supplementary Table 3**. Expression data for CRISPR activation experiments (Excel file). Contains normalized log2 expression values, fold changes, p-values, FDR-adjusted p-values, gene annotations, and per-sample expression values for all detected transcripts. A separate sheet contains the top differentially expressed genes upon TEDAR activation in differentiated keratinocytes.

**Supplementary Table 4**. Top 10 GO Biological Process terms enriched in TEDAR-regulated genes after CRISPR-mediated activation in differentiated keratinocytes (Excel file).

**Supplementary Table 5**. Expression data for TEDAR knockdown experiments (Excel file). Contains normalized log2 expression values, fold changes, p-values, FDR-adjusted p-values, gene annotations, and per-sample expression values for all detected transcripts. A separate sheet contains the top differentially expressed genes in TEDAR-depleted 3D organotypic skin cultures.

**Supplementary Table 6**. Top 10 GO Biological Process terms enriched in TEDAR-depleted 3D organotypic skin cultures (Excel file).

**Supplementary Table 7**. List of TEDAR-interacting proteins detected by mass spectrometry following RNA pulldown (Excel file).

**Supplementary Table 8**. Primers used for RT-qPCR amplification (Excel file).

**Supplementary Table 9**. List of antibodies and plasmids used in the study (Excel file).
